# CD40 transcriptomic expression patterns across malignancies: implications for clinical trials of CD40 agonists

**DOI:** 10.1007/s00262-025-04197-8

**Published:** 2025-11-03

**Authors:** Yuji Uehara, Daisuke Nishizaki, Yu Fujiwara, Hirotaka Miyashita, Suzanna Lee, Sarabjot Pabla, Paul DePietro, Heidi Ko, Taylor J. Jensen, Jason K. Sicklick, Shumei Kato, Razelle Kurzrock

**Affiliations:** 1 Department of Experimental Therapeutics, National Cancer Center Hospital, 5 Chome-1-1 Tsukiji, Chuo-Ku, Tokyo, 104-0045 Japan; 2 Department of Thoracic Oncology and Respiratory Medicine, Tokyo Metropolitan Cancer and Infectious Diseases Center, Komagome Hospital, Tokyo, Japan; 3 Center for Personalized Cancer Therapy and Division of Hematology and Oncology, Department of Medicine, University of California San Diego (UCSD), Moores Cancer Center, 3855 Health Sciences Drive, La Jolla, CA 92093 USA; 4 Department of Medicine, Roswell Park Comprehensive Cancer Center, Buffalo, NY USA; 5 Department of Oncology, Dartmouth Cancer Center, Lebanon, NH USA; 6 Labcorp, Buffalo, NY USA; 7 Labcorp, Durham, NC USA; 8 Division of Surgical Oncology, Department of Surgery, Center for Personalized Cancer Therapy, University of California San Diego, La Jolla, CA USA; 9 MCW Cancer Center and Genomic Sciences and Precision Medicine Center, Medical College of Wisconsin, Milwaukee, WI USA; 10 Department of Oncology, MCW Cancer Center, Milwaukee, WI USA; 11 Department of Oncology, University of Nebraska, Omaha, NE USA

**Keywords:** TNFRSF5, Biomarkers, Clinical trials, Immune checkpoints, Combination therapy

## Abstract

**Background:**

CD40 is a T-cell co-stimulatory receptor targeted by next-generation immunotherapies. We conducted a pan-cancer transcriptome analysis of CD40, its ligand, and related immune markers to evaluate co-expression patterns and clinical outcomes.

**Methods:**

We analyzed transcriptome data for CD40, its ligand, and other common checkpoints and co-stimulators (PD-1, PD-L1, PD-L2, CTLA-4, LAG-3, ICOS, CD27, CD28, OX40, and GITR). RNA expression was classified as high (75–100th percentile), moderate (25–74th), or low (0–24th) against a reference population of 735 previously tested solid tumors.

**Results:**

Of 514 patients, 114 (22%) showed high, 247 (48%) moderate, and 153 (30%) low CD40 RNA expression. High CD40 expression was most frequent in liver and bile duct (42%), pancreatic (42%), and ovarian (40%) cancers. Both high CD40 and low–moderate CD40 ligand expression—potentially conducive to CD40 agonist therapy—was most frequent in ovarian (33%) and pancreatic (24%) cancer. In both UCSD (N = 514) and TCGA (N = 10,953) cohorts, high CD40 expression significantly correlated with high CD28 and GITR. High CD40 RNA levels were not prognostic for overall survival (OS) from metastatic disease (*P* = 0.2) (n = 272 immune checkpoint inhibitor (ICI)-naïve patients). High CD40 expression correlated with longer OS from immunotherapy initiation (n = 217 ICI-treated patients; *P* = 0.04, univariable analysis), but not multivariable analysis, suggesting it may not be an independent predictive biomarker.

**Conclusion:**

High CD40 expression correlated with liver and bile duct, pancreatic, and ovarian cancers, as well as with CD28 and GITR transcripts. Immune marker co-expression in individual patients merits further exploration for the development of CD40-based and other immunotherapy interventions.

**Supplementary Information:**

The online version contains supplementary material available at 10.1007/s00262-025-04197-8.

## Introduction

Immunotherapy has transformed cancer treatment. While most advanced approaches focus on immune checkpoint inhibitors (ICIs) such as PD-1, PD-L1, CTLA-4, and LAG-3 antibodies, it is recognized that both co-stimulatory and co-inhibitory pathways are essential for T-cell activation. Consequently, researchers are investigating T-cell co-stimulatory receptors as targets for next-generation cancer immunotherapies, although significant clinical efficacy remains elusive.

**Fig. 1:**
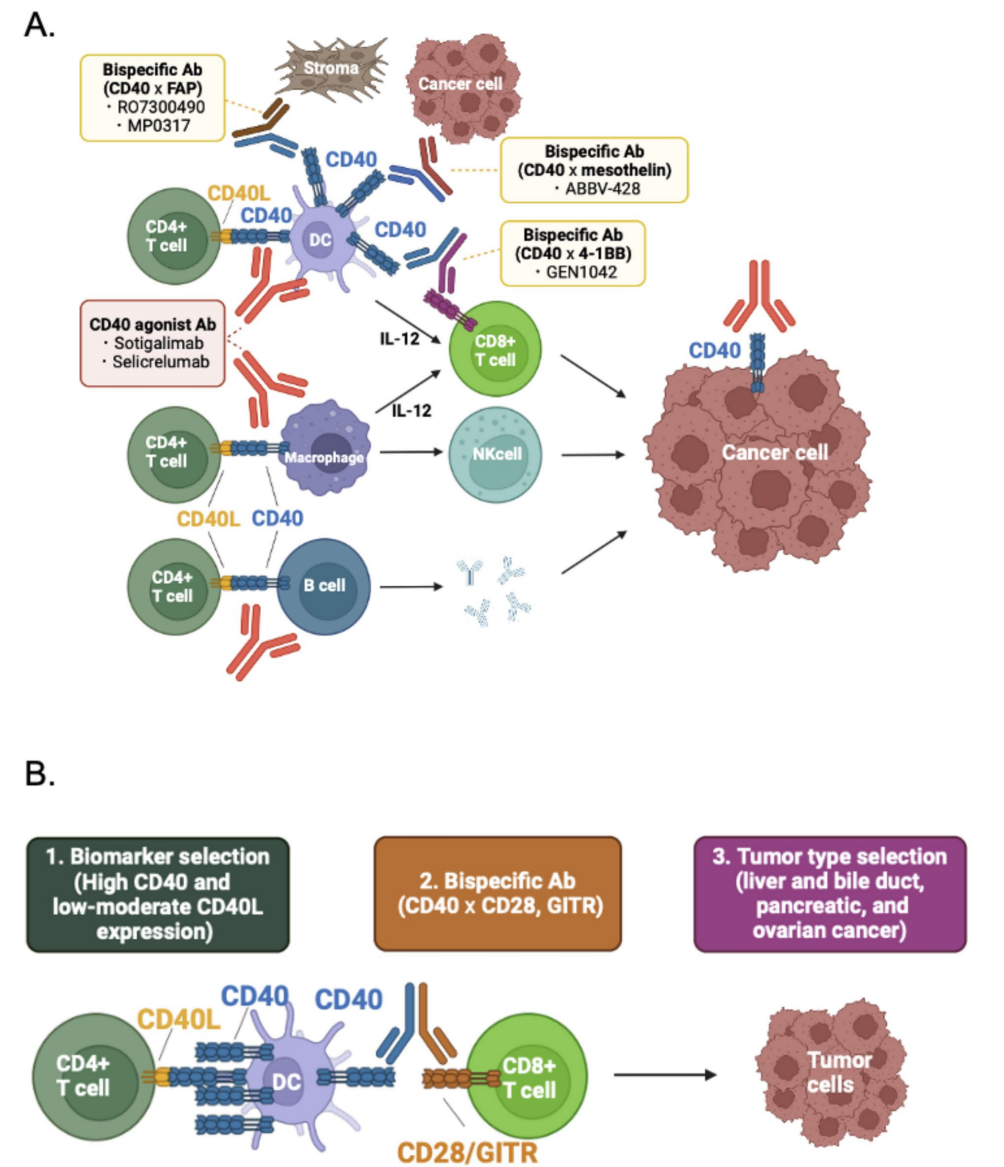
Effects of CD40–CD40L signaling and potential strategies involving CD40 agonistic drugs. **A** CD40L is expressed on CD4⁺ T-cells. The binding of CD40 to CD40L activates dendritic cells (DCs), macrophages, and B cells, enhancing their ability to process and present tumor-associated antigens (TAAs) to cytotoxic CD8⁺ T-cells. In macrophages, CD40–CD40L interactions lead to the production of tumor necrosis factor-alpha (TNF-α) and facilitate antibody-dependent cellular cytotoxicity (ADCC). These interactions in DCs and macrophages also result in the secretion of interleukin 12 (IL-12), which is crucial for natural killer (NK) cell-mediated antitumor activity. CD40–CD40L activation in B cells underpins T-cell immunity and antibody production. Additionally, CD40 activation can directly induce apoptosis in cancer cells. Signaling through CD40 agonists upregulates co-stimulatory molecules on DCs, thereby enhancing their capacity to present antigens and activate T-cells. CD40 agonists can also reprogram tumor-associated macrophages (TAMs) from a tumor-promoting phenotype to an activated, antitumor state. Furthermore, bispecific antibodies targeting TAAs (e.g., mesothelin) on tumor cells or fibroblast activation protein (FAP) on stromal cells can cross-link CD40 agonists, thereby stimulating antitumor immunity within the tumor microenvironment. **B** A viable approach to enhance antitumor efficacy involves the use of CD40 agonistic drugs, possibly in combination with bispecific antibodies, to amplify immune responses against the tumor. The figure was prepared in BioRender (https://www.biorender.com/). Abbreviations: ADCC, antibody-dependent cellular cytotoxicity; CD40, cluster of differentiation 40; CD40L, CD40 ligand; DC, dendritic cell; FAP, fibroblast activation protein; IL-12, interleukin 12; NK cell, natural killer cell; TAA, tumor-associated antigen; TAM, tumor-associated macrophage; TNF-α, tumor necrosis factor-alpha

CD40 (TNFRSF5), a member of the tumor necrosis factor receptor (TNFR) superfamily, is one of the key co-stimulatory molecules under study. Its ligand, CD40L (also known as CD154 or TNFSF5), is expressed on CD4⁺ T-cells [1, 2]. Upon binding, CD40–CD40L activates dendritic cells (DCs), macrophages, and B cells, enhancing their capacity to process and present tumor-associated antigens (TAAs) to cytotoxic CD8⁺ T-cells (Fig. 1A). In macrophages, this interaction triggers the production of tumor necrosis factor-alpha (TNF-α) and facilitates antibody-dependent cellular cytotoxicity (ADCC). It also prompts DCs and macrophages to secrete interleukin 12 (IL-12), a key factor in natural killer (NK) cell-mediated antitumor activity.

Studies targeting CD40 aim to enhance anti-cancer immune responses (Fig. 1A, Supplementary Table S1). CD40 agonists upregulate co-stimulatory molecules by activating dendritic cells, thereby improving antigen presentation and T-cell activation. Bispecific antibodies that target tumor antigens, fibroblast activation protein on stromal cells, or other receptors such as 4–1BB can also cross-link CD40 agonists, promoting antitumor immunity in the tumor microenvironment [3]. These agonists, used alone or in combination with immune checkpoint inhibitors (ICIs) or other monoclonal antibodies, have been investigated to improve both safety and efficacy [1, 4]. However, results from these clinical trials—particularly in solid tumors—have been modest (Supplementary Table S1). One possible explanation for this limited efficacy is that most reported and ongoing CD40 agonist trials lack biomarker-based patient selection. Several studies suggest that incorporating transcriptomic analysis, alongside genomic data, could help identify each tumor’s unique immunologic signature and enable more personalized treatment strategies [5–11].

Herein, we examine the CD40 transcriptomic landscape in 514 patients, including 489 individuals with advanced or metastatic cancers and available clinical curation. We discuss the potential therapeutic implications arising from these observed patterns and their inherent heterogeneity.

## Materials and methods

### Patients

Altogether, 489 patients with curated clinical data (out of 514 patients with tissue samples) were included from the UCSD Moores Cancer Center for Personalized Therapy. Patients were treated for various solid tumors; for those who had > 1 sample taken on different dates, only the earliest sample was used. The cohort comprised patients with advanced or metastatic disease. An analysis of the biopsy location showed that 84% (429/514) of samples were from metastatic sites, while 16% (85/514) were from primary tumors (Supplemental Table S2). Clinical data were collected from electronic records. This study followed the guidelines of the IRB-approved UCSD-Profile Related Evidence Determining Individualized Cancer Therapy (PREDICT) study (NCT02478931, https://clinicaltrials.gov/ct2/show/NCT02478931); patients gave written consent for any investigational interventions, as detailed previously [8–10, 12–16]. This study was conducted in accordance with the principles of the Declaration of Helsinki.

### Tissue collection and analysis of cancer immunity markers

Formalin-fixed, paraffin-embedded (FFPE) tumors were processed at a CLIA/CAP-certified laboratory (OmniSeq) and underwent RNA sequencing. Total RNA was extracted (truXTRAC FFPE extraction kit (Covaris)). RNA expression was measured with Oncomine Immune Response Research Assay (OIRRA) using the immuneResponseRNA (v5.2.0.0) plugin on the Torrent Suite. Of these, 12 immune-related genes (CD40, PD-1, PD-L1, PD-L2, CTLA-4, LAG-3, ICOS, 4–1BB, CD27, CD28, OX40, and GITR) were emphasized (Supplementary Table S3). The gene list for each panel is summarized in Supplementary Table S4. Transcript abundance was converted to percentile ranks (0–100) against a reference population of 735 pretreated, pan-cancer solid tumor samples [17]. This diverse cohort, processed at a single CLIA/CAP-certified laboratory (OmniSeq), spanned 35 tumor histologies, various disease stages, and patient demographics, representing a real-world distribution of immune gene expression. To ensure comparability across sequencing runs, raw transcript reads were normalized to normalized reads per million (nRPM) values. (This process involved two key steps: (1) Background subtraction: for each transcript, the absolute read counts from a no-template control (NTC) were considered background and subtracted from the absolute read counts of that same transcript in every other sample within the same batch. (2) Housekeeping gene normalization: the background-subtracted reads for 10 constitutively expressed housekeeping (HK) genes were compared against a predetermined, stable HK RPM profile established from multiple sequencing runs. The median ratio of this comparison was calculated to create a sample-specific 'Normalization Ratio'.) The final nRPM for any given gene was calculated by dividing its background-subtracted read count by this Normalization Ratio. Based on this normalized ranking, 'high expression' for an immune response gene was defined as a value at or above the 75th percentile, corresponding to the top quartile of the reference population. The definitions for moderate (25th–74th percentile) and low (0–24th percentile) gene expression were established in the same manner [8–10, 12–15]. This normalization was applied to the UCSD cohort; TCGA data were analyzed using their standardly available normalized expression values. The assessment of PD-L1 protein expression, as well as tumor mutational burden (TMB) and microsatellite instability (MSI), was described in previous publications [8–10, 12–15].

### Analysis of TCGA PanCancer atlas cohort from cBioPortal

For a total of 10,953 patients across 32 cancer types (Supplementary Table S5), RNA expression levels were analyzed from the Pan-Cancer The Cancer Genome Atlas (TCGA) Atlas cohort to evaluate correlations between CD40 and other gene expressions or genomic alterations, using cBioPortal (cBio cancer genomics portal, https://www.cbioportal.org).

### Clinical endpoints and statistical analyses

Associations between high CD40 expression, cancer types that had 15 or more samples and/or a higher proportion of patients with high CD40 expression than the average, and immune markers were examined (univariable logistic regression). Variables with *P* values ≤ 0.05 were entered into multivariable logistic regression. For variables with missing data (TMB and MSI), multiple imputation was implemented under the missing at random assumption [18].

Differential gene expression (DEG) analysis was performed to identify significantly expressed genes between the CD40-high and CD40-low/moderate groups using the R package limma (version 3.58.1). The results were visualized using a volcano plot created with the R package ggplot2 (version 3.5.1) and a heatmap generated with the pheatmap package (version 1.0.12). Differentially expressed genes were defined as those with an adjusted *P* value < 0.05 and an absolute log2 fold change ≥ 0.5.

To identify genes and pathways with differential mutation patterns based on CD40 expression, we first stratified samples into CD40-high and CD40-low/moderate cohorts using RNA expression data. With these cohorts defined, we performed an enrichment analysis on somatic mutation data at both the individual gene level and the pathway level, utilizing the 50 MSigDB Hallmark gene sets [19]. A Fisher's Exact Test was applied to each gene and pathway to calculate a *P* value and odds ratio. To account for multiple comparisons, we adjusted the raw *P* values using the Benjamini–Hochberg procedure to generate q-values. Features were deemed significantly enriched if they met a false discovery rate threshold of q < 0.05 and an absolute log2 odds ratio greater than 1. The results from both the gene and pathway analyses were visualized as volcano plots.

Kaplan–Meier analyses were used to evaluate overall survival (OS) from the time of diagnosis of metastatic/locally advanced disease and, for immunotherapy-treated patients, OS and progression-free survival (PFS) were assessed from therapy initiation. Survival outcomes were compared between those with high (≥ 75th rank) vs. moderate/low (< 75th rank) CD40 RNA expression using the log-rank test. Factors influencing OS and PFS were subsequently examined with multivariable Cox proportional hazard models. The data cut-off for analysis was June 24, 2022; a *P* value ≤ 0.05 was deemed significant. Patients still alive (for OS) or progression-free (for PFS) at the time of last contact (or at data cut-off, whichever came first) were censored at that time. Statistical analyses were conducted using R version 4.3.1.

## Results

### Patient characteristics

We evaluated 514 tumors representing 31 different cancer types (Supplementary Table S2); 489 patients with advanced/metastatic disease had evaluable clinical correlative data (Supplementary Fig. S1). The median patient age was 61 years; 310 (60%) were women. The most frequent tumor types were colorectal cancer (n = 140), pancreatic cancer (n = 55), and breast cancer (n = 49).

### CD40 transcript expression varies across and within cancer types, but is high in liver and bile duct, pancreatic, and ovarian cancers

**Fig. 2:**
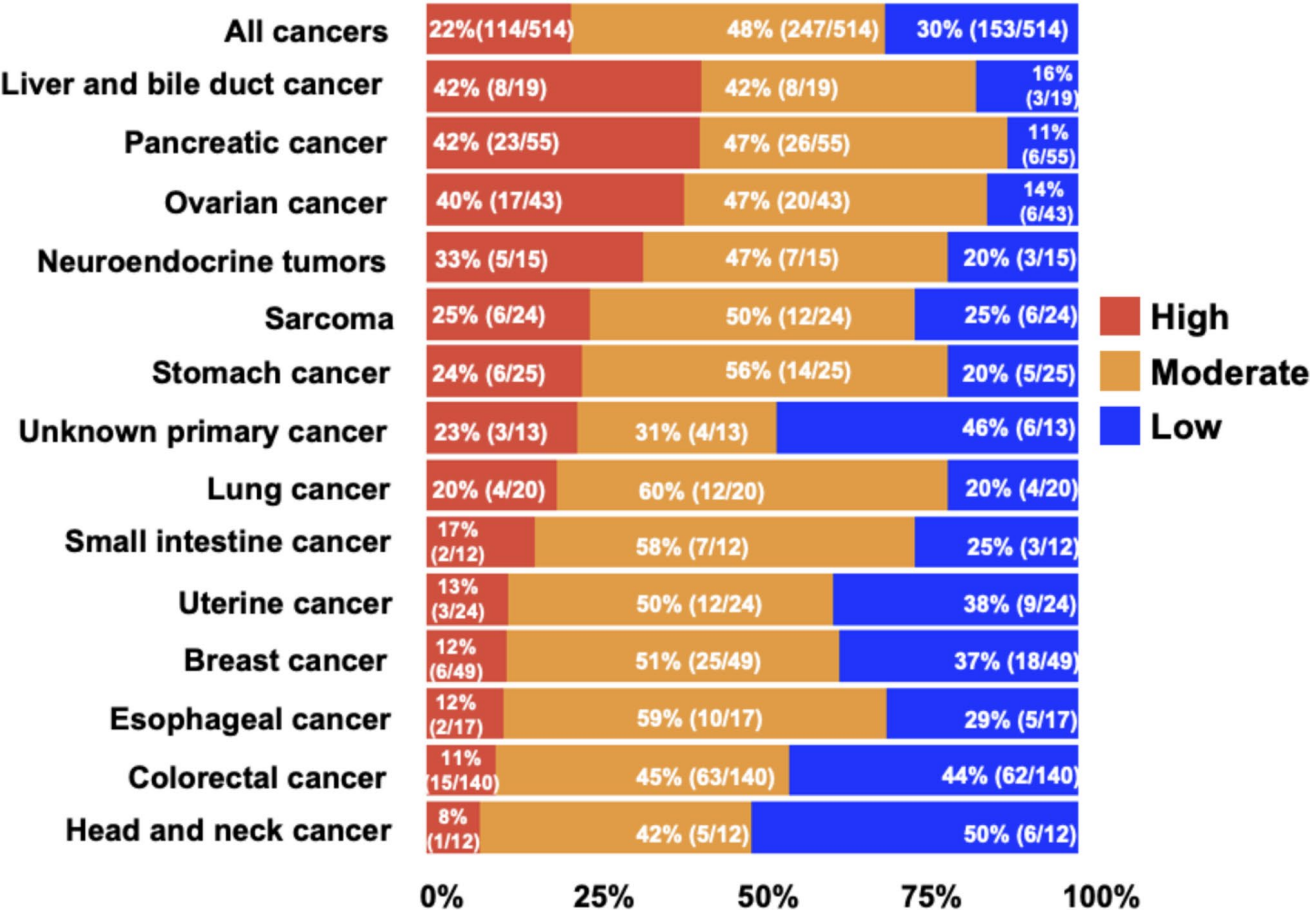
Proportion of patients with high versus moderate or low CD40 expression (N = 514). Proportion of patients with high, moderate, or low RNA expression of CD40, stratified by cancer type. Under “All Cancers,” all patients were included; for each specific cancer type, only those with more than 10 representative samples were included. High RNA expression of CD40 was observed in liver and bile duct cancer (42% [8/19]), pancreatic cancer (42% [23/55]), ovarian cancer (40% [17/43]), neuroendocrine tumors (33% [5/15]), sarcoma (25% [6/24]), stomach cancer (24% [6/25]), and unknown primary cancer (23% [3/13]). All of these exceeded the average proportion (22%) across all cancers. Definition of RNA expression: High = 75–100th percentile RNA rank: moderate = 25–74th; and low = 0–24th percentile rank value score (see Methods)

CD40 RNA expression varied across different cancer types (Fig. 2). CD40 expression was classified as “high” (75–100th percentile), “moderate” (25–74th percentile), or “low” (0–24th percentile). Among all samples (N = 514), 114 (22%) had high RNA CD40 expression. Liver and bile duct cancers, pancreatic cancer, and ovarian cancer most frequently had high CD40 RNA expression (42%, 42%, and 40% of tumors, respectively). In contrast, colorectal and head and neck cancers had the lowest frequencies of high CD40 RNA expression (11% and 8% of tumors, respectively).

In multivariable analysis, high CD40 RNA expression was significantly associated with liver and bile duct (odds ratio [OR] 5.4, 95% confidence interval [CI] 1.9–15.0, *P* < 0.001), pancreatic (OR 4.5, 95% CI 2.3–8.8, *P* < 0.001), and ovarian (OR 4.4, 95% CI 2.1–9.2, *P* < 0.001) cancers (Table 1).

**Table 1:** Clinical and immune characteristics associated with high CD40 RNA expression (≥ 75th percentile RNA rank) (N = 514)

	Characteristics		Univariable		Multivariable	
Name	Condition	Proportion of tumors with high CD40 expression	Odds ratio (95% CI)	*P* value	Odds ratio (95% CI)	*P* value
Age (years)	≥ 61	25% [63/256]	1.3 (0.87–2.0)	0.19		
	< 61	20% [51/258]	–			
Sex	Male	19% [39/204]	0.74 (0.48–1.1)	0.18		
	Female	24% [75/310]	–			
Liver and bile duct cancer	Yes	42% [8/19]	2.7 (1.0–6.8)	0.040	5.4 (1.9–15.0)	** < 0.001**
	No	21% [106/495]	–			(Correlated with higher level of CD40)
Pancreatic cancer	Yes	42% [23/55]	2.9 (1.6–5.2)	< 0.001	4.5 (2.3–8.8)	** < 0.001**
	No	20% [91/459]	–			(Correlated with higher level of CD40)
Ovarian cancer	Yes	40% [17/43]	2.5 (1.3–4.8)	0.005	4.4 (2.1–9.2)	** < 0.001**
	No	21% [97/471]	–			(Correlated with higher level of CD40)
Neuroendocrine tumors	Yes	33% [5/15]	1.3 (0.55–5.1)	0.30		
	No	22% [109/499]	–			
Sarcoma	Yes	25% [6/24]	1.2 (0.41–2.9)	0.73		
	No	22% [106/490]	–			
Stomach cancer	Yes	24% [6/25]	1.1 (0.40–2.7)	0.82		
	No	22% [108/489]	–			
Unknown primary cancer	Yes	23% [3/13]	1.1 (0.23–3.5)	0.94		
	No	22% [111/501]	–			
MSI^a^	Unstable	13% [2/15]	0.53 (0.08–2.0)	0.40		
	Stable	23% [105/465]	–			
TMB (mutations/Mb)^b^	≥ 10	6.1% [2/33]	0.31 (0.04–1.02)	0.051		
	< 10	22% [90/417]	–			
PD-L1 IHC^c^	Positive (CPS ≥ 1)	20% [31/156]	1.6 (1.04–2.5)	0.031	1.2 (0.68–2.1)	0.52
	Negative	13% [46/358]	–			
PD-1 RNA level	High	47% [44/93]	4.5 (2.8–7.3)	< 0.001	1.8 (0.88–3.7)	0.10
	Low/Moderate	17% [70/421]	–		–	
PD-L1 RNA level	High	45% [30/67]	3.5 (2.0–6.0)	< 0.001	1.7 (0.76–3.7)	0.19
	Low/Moderate	19% [84/447]	–			
PD-L2 RNA level	High	38% [38/100]	2.8 (1.7–4.4)	< 0.001	1.1 (0.56–2.1)	0.81
	Low/Moderate	18% [76/414]	–		–	
CTLA-4 RNA level	High	38% [33/87]	2.6 (1.6–4.3)	< 0.001	0.5 (0.21–1.1)	0.10
	Low/Moderate	19% [81/427]	–		–	
LAG-3 RNA level	High	41% [47/116]	3.4 (2.1–5.3)	< 0.001	1.8 (0.97–3.3)	0.054
	Low/Moderate	17% [67/398]	–		–	
ICOS RNA level	High	43% [30/70]	3.2 (1.8–4.3)	< 0.001	0.7 (0.30–1.7)	0.44
	Low/Moderate	19% [84/444]	–		–	
4-1BB RNA level	High	45% [35/77]	3.8 (2.3–6.3)	< 0.001	1.5 (0.75–3.1)	0.23
	Low/Moderate	10% [42/437]	–		–	
CD27 RNA level	High	43% [42/98]	3.6 (2.2–5.7)	< 0.001	1.4 (0.68–2.8)	0.36
	Low/Moderate	17% [72/416]	–		–	
CD28 RNA level	High	42% [43/102]	3.5 (2.2–5.6)	< 0.001	2.5 (1.2–4.9)	**0.009**
	Low/Moderate	17% [71/412]	–		–	(High CD28 correlated with higher level of CD40)
OX40 (TNFRSF4) RNA level	High	37% [45/122]	2.7 (1.7–4.3)	< 0.001	1.5 (0.82–2.6)	0.19
	Low/Moderate	18% [69/392]	–		–	
GITR (TNFRSF18) RNA level	High	39% [39/99]	2.9 (1.8–4.8)	< 0.001	2.1 (1.1–3.8)	**0.017**
	Low/Moderate	18% [75/415]	–		–	(High GITR correlated with higher level of CD40)

Bold values indicate statistical significance (*P* value ≤ 0.05)

Abbreviations: CI, confidence interval; IHC, immunohistochemistry; Mb, megabase; MSI, microsatellite instability; TMB, tumor mutation burden

^a^MSI was available in 480 patients

^b^Among 514 patients, TMB was available in 450 patients

^c^One sample for PD-L1 IHC was missing and was considered < 1

Variables with *P* ≤ 0.05 from univariable analyses were included for multivariable analysis

For variables with missing data (MSI and TMB), a multiple imputation was implemented

### Landscape of high CD40 expression and low-moderate CD40 ligand expression

**Fig. 3:**
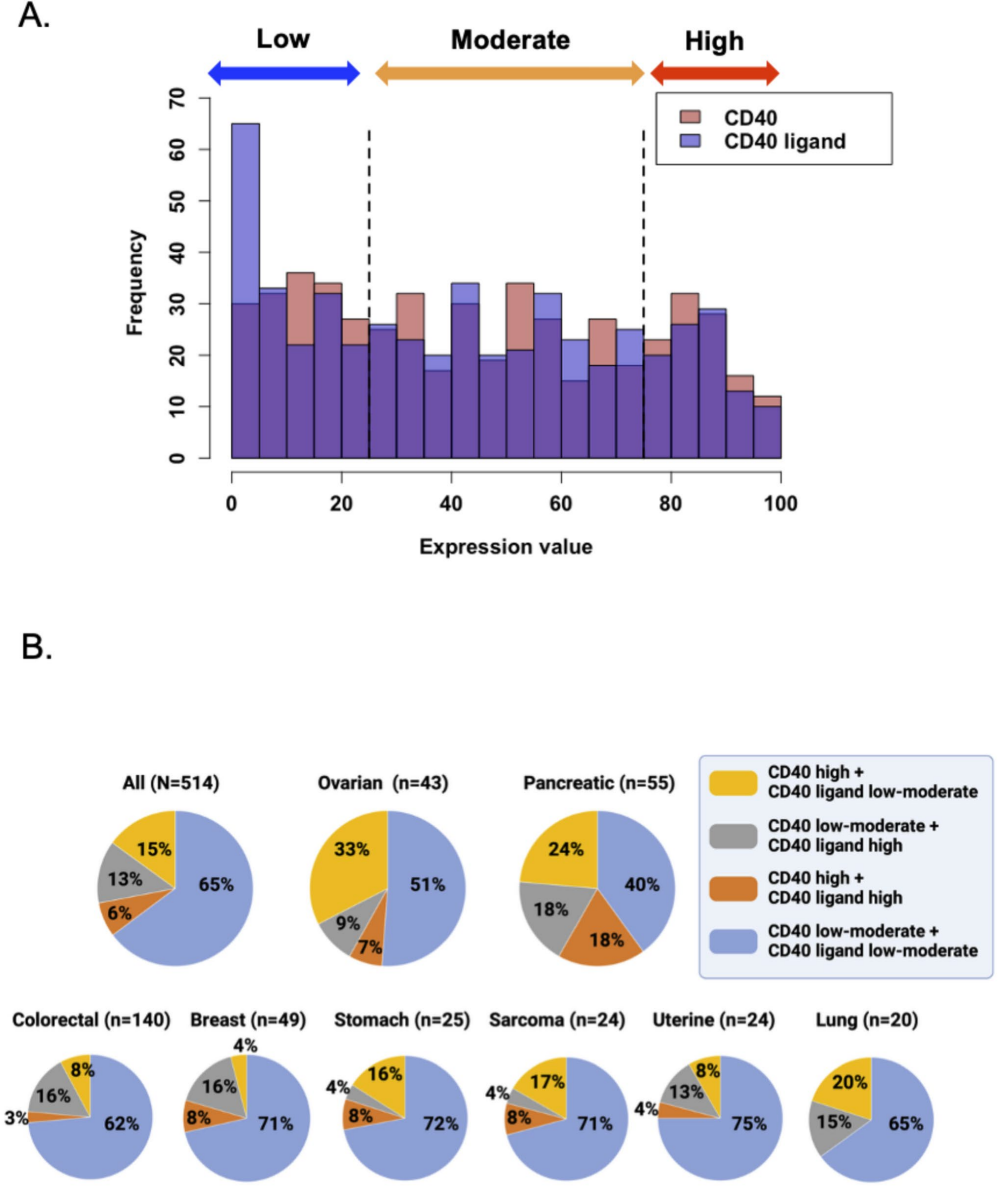
Proportion of patients with different combinations of high versus low–moderate CD40/CD40 ligand (CD40L) RNA expression (N = 514). **A** Overlaid histograms illustrating the frequency distribution of expression values for CD40 (red) and its ligand, CD40L (blue). Definition of RNA expression: High = 75–100th percentile RNA rank: moderate = 25–74th; and low = 0–24th percentile rank value score (see Methods). **B** All malignancies and those with ≥ 15% of patients showing high CD40 RNA expression and low–moderate CD40L expression. All other malignancies with ≥ 20 samples. CD40 RNA expression in the 75th–100th percentile was defined as high, an CD40L expression in the 0–74th percentile was defined as low–moderate. The figure was prepared in BioRender (https://www.biorender.com/). Abbreviations: CD40, cluster of differentiation 40; CD40L, cluster of differentiation 40 ligand

We first examined the overall distribution of CD40 and CD40 ligand (CD40L) expression values across the patient cohort (Fig. 3A). The frequency histogram revealed that CD40L expression was predominantly low, with a large peak at the lowest end of the expression scale. In contrast, CD40 receptor expression was more broadly distributed across the low, moderate, and high ranges. Based on these categories, we evaluated the proportion of patients who had high CD40 expression and low-moderate CD40L expression in malignancies with more than 20 representative samples. From a biological perspective, high receptor expression coupled with low ligand expression may be most amenable to treatment with CD40 agonists. Ovarian and pancreatic cancers had the highest proportions of patients exhibiting both high CD40 and low-moderate CD40L expression—33% (14/43) and 24% (13/55), respectively—representing the only two malignancies in which more than 20% of patients had this expression profile (Fig. 3B).

In comparison, the proportions of patients who had high CD40 expression and low-moderate CD40L expression were 20% (4/20) in lung, 17% (4/24) in sarcoma, 16% (4/25) in stomach, 8% (2/24) in uterine, 8% (11/140) in colorectal, and 4% (2/49) in breast (Fig. 3B). Overall, there was notable variability in expression patterns both within and between tumor types.

### High CD40 RNA levels correlate with specific immune markers (CD28 and GITR) in the UCSD (n = 514) and TCGA PanCancer atlas (n = 10,953) cohorts

**Fig. 4:**
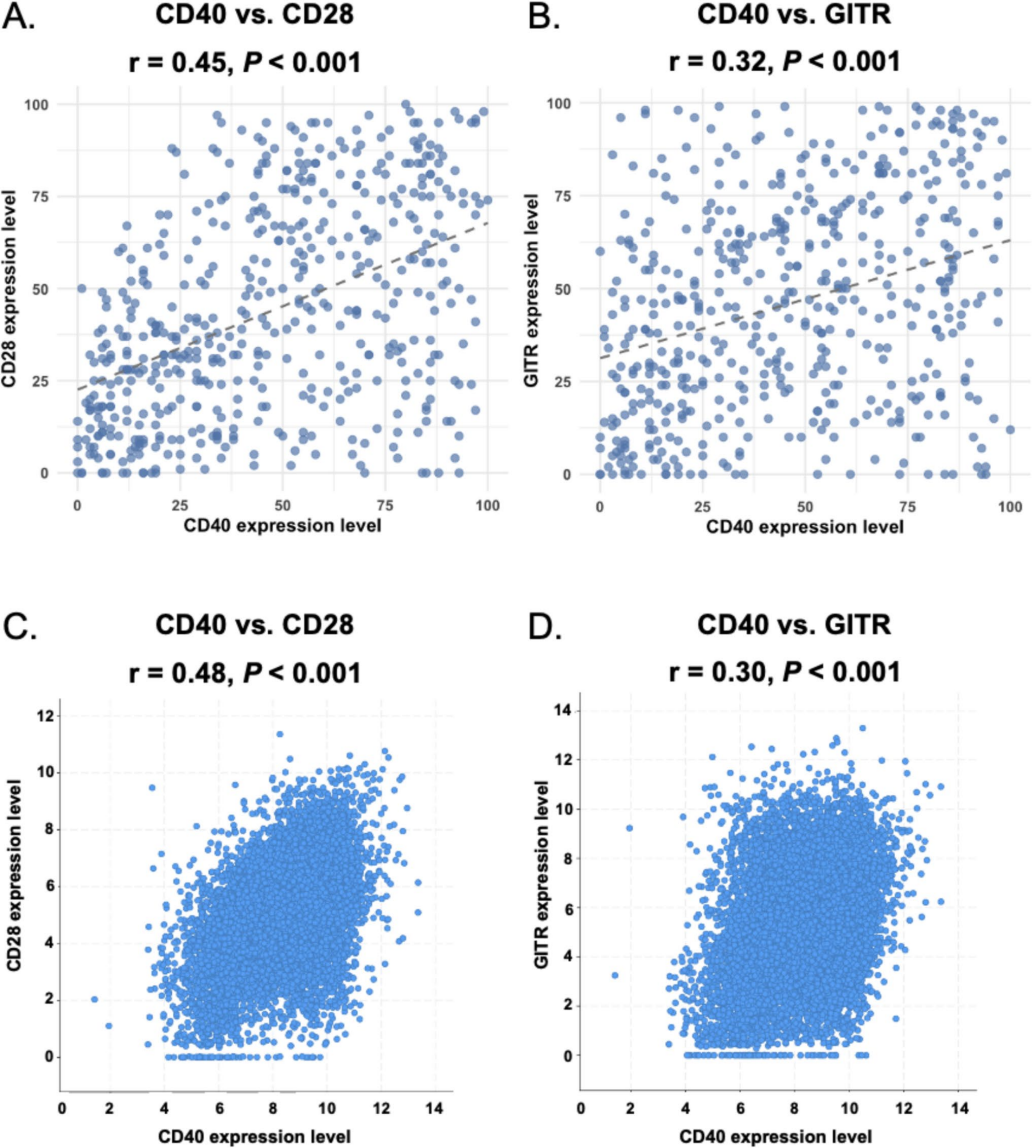
Associations between CD40 expression and immune markers in the UCSD (N = 514) and TCGA PanCancer Atlas Cohorts (N = 10,953). **A** Scatter plots of CD40 versus CD28 and GITR in the UCSD cohort (n = 514). For CD40 versus CD28 mRNA expression, Spearman’s rho = 0.45 (*P* < 0.001). **B** Scatter plot of CD40 versus GITR mRNA expression. Spearman’s rho = 0.32 (P < 0.001). **C** Scatter plots of CD40 versus CD28 and GITR in the TCGA PanCancer Atlas studies (n = 10,953). For CD40 versus CD28 mRNA expression (RSEM), Spearman’s rho = 0.48 (*P* < 0.001). **D** Scatter plot of CD40 versus GITR mRNA expression (RSEM). Spearman’s rho = 0.30 (*P* < 0.001). Abbreviations: CD28, cluster of differentiation 28; CD40, cluster of differentiation 40; GITR, glucocorticoid-induced tumor necrosis factor receptor; RSEM, RNA-Seq by Expectation–Maximization; TCGA, The Cancer Genome Atlas

We examined 12 genes associated with immunomodulatory molecules related to the TNF receptor superfamily or other co-stimulatory molecules, as well as immune checkpoint inhibitors (ICIs) used in clinical settings: 4-1BB, PD-1, PD-L1, PD-L2, CTLA-4, LAG-3, ICOS, CD40, CD27, CD28, OX40, and GITR (Supplementary Table S3, Table 1). Among immune markers, high CD40 RNA expression was significantly associated with CD28 (OR 2.5, 95% CI 1.2–4.9, *P* = 0.009) and GITR (OR 2.1, 95% CI 1.1–3.8, *P* = 0.017) in the multivariable analysis. In the scatter plot, CD28 RNA expression was significantly correlated with CD40 (Spearman correlation coefficient: 0.45, *P* < 0.001; Fig. 4A). Additionally, GITR (a co-stimulatory immune checkpoint expressed on the surface of many immune cells) expression was also significantly correlated with CD40 (Spearman correlation coefficient: 0.32,* P* < 0.001; Fig. 4B). (CD28 is a receptor that binds to CD80 [B7.1] and CD86 [B7.2] proteins on antigen-presenting cells [APCs]. CD28 and CD40 are both co-stimulatory molecules involved in T-cell activation, but they function in a coordinated manner with CD28 primarily initiating the T-cell response while CD40, expressed on APCs, helps sustain/amplify the immune response by providing additional signals for T-cell activation and differentiation, particularly in B cell interactions [20]).

In the TCGA PanCancer Atlas (n = 10,953) cohort, CD28 RNA expression was significantly correlated with CD40 (Spearman correlation coefficient: 0.48, *P* < 0.001; Fig. 4C). Additionally, GITR expression was also significantly correlated with CD40 (Spearman correlation coefficient: 0.30,* P* < 0.001; Fig. 4D).

### Immune checkpoint targeted by FDA-approved ICIs are enriched in the high CD40 group

**Fig. 5:**
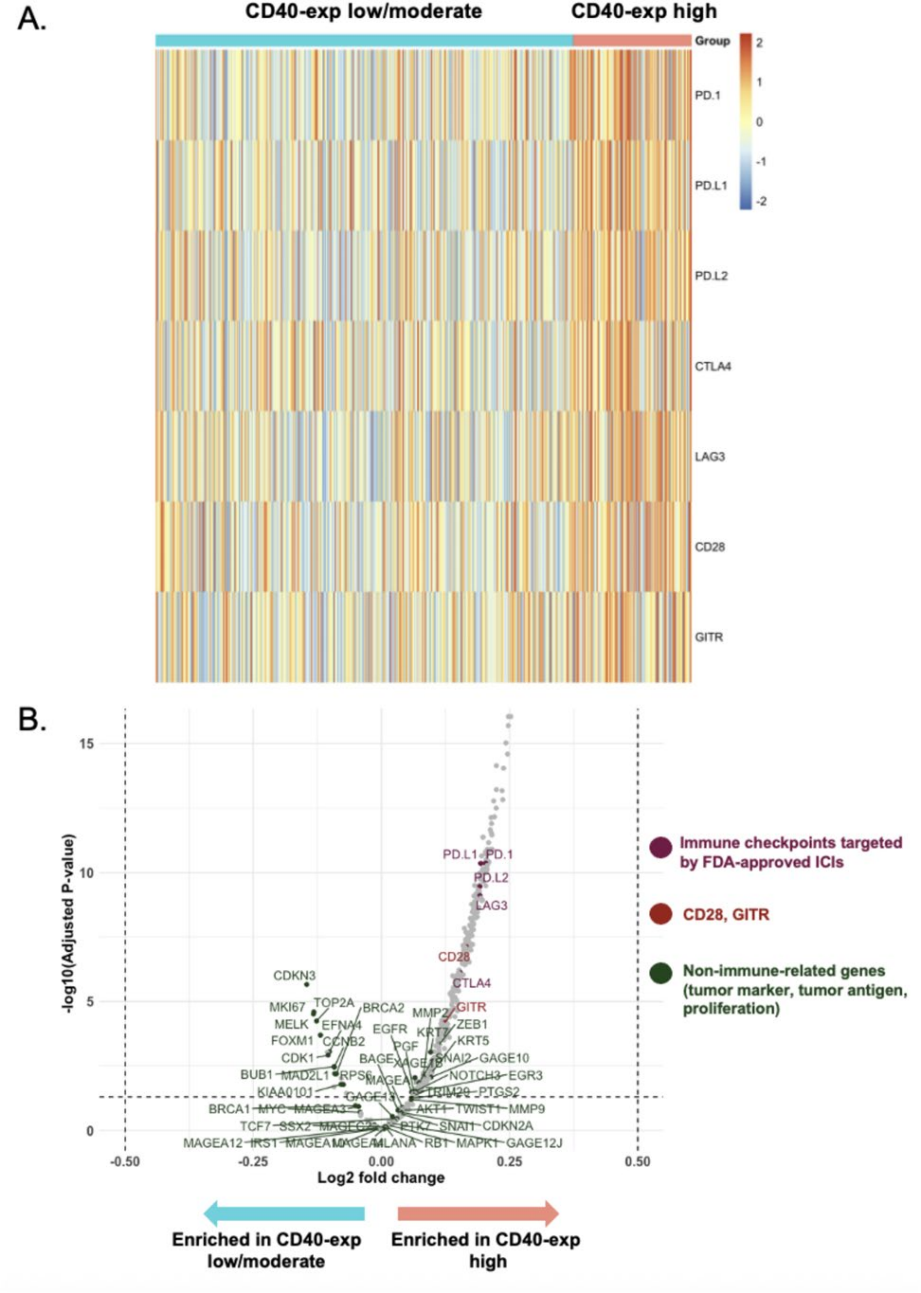
Correlation of high CD40 expression with an enriched immune checkpoint signature (N = 514). **A** Heatmap of key immune checkpoint and co-stimulatory molecule expression in patient samples, grouped by low/moderate versus high CD40 expression. Each column represents a patient sample, and each row represents a gene. Color corresponds to the expression z-score, with red indicating higher and blue indicating lower expression. PD-1, PD-L1, PD-L2, CTLA4, and LAG3 show higher expression in the high CD40 expression group. **B** Volcano plot from the differential gene expression analysis comparing the high-CD40 group to the combined low/intermediate-CD40 groups. The x-axis represents the log2 fold change, and the y-axis represents the -log10 adjusted *p* value. Genes enriched in the high-CD40 group are on the right. Immune checkpoints targeted by FDA-approved ICIs (purple) are upregulated, whereas non-immune-related genes for tumor markers, antigens, and proliferation (green) are not. The horizontal dashed line indicates the threshold for statistical significance (adjusted *P* value < 0.05 and log2 fold change ≥ 0.5). Abbreviations: CTLA-4, cytotoxic T-lymphocyte-associated protein 4; FDA, Food and Drug Administration; ICIs, immune checkpoint inhibitors; LAG-3, lymphocyte-activation gene 3; PD-1, programmed cell death protein 1; PD-L1, programmed death-ligand 1; PD-L2, programmed death-ligand 2

The heatmap revealed distinct expression patterns for immune checkpoints targeted by FDA-approved ICIs (PD-1, PD-L1, PD-L2, CTLA-4, and LAG-3). These expression was enriched in the high-CD40 group (Fig. 5A). To verify the biological specificity of this association, a differential gene expression analysis was performed between the high-CD40 group and the combined low/intermediate-CD40 groups. This analysis confirmed that while key immune checkpoint transcripts were enriched in the high-CD40 group, non-immune-related genes (tumor markers, antigens, and proliferation; Supplementary Table S4) were not (Fig. 5B). These findings suggest that the co-expression of these checkpoints constitutes a specific immune signature rather than a general increase in transcriptional activity. While this relationship between CD40 and immune checkpoints was observed in the univariable analysis, it did not remain significant in our multivariable model (Table 1).

### High CD40 expression is associated with increased CD4 and CD8 T-cell gene signatures

To investigate the relationship between the CD40 signaling pathway and T-cell infiltration, we analyzed RNA expression of CD40, CD4, and CD8 in a cohort of 514 samples (Table 2). Patients with high CD40 expression were substantially more likely to exhibit high expression of CD4, CD8, and a combined CD4/8 signature compared to those with low or moderate CD40 expression. Specifically, 52% of patients with high CD40 expression (59/114) had a high CD4/8 expression signature, more than double the rate observed in the low/moderate CD40 group (23%; 91/400) (*P* < 0.001).

**Table 2:** Relationship between CD40 expression and CD4/CD8 expression (N = 514)

RNA expression^a^	High CD4 expression (n = 107)	High CD8 expression (n = 89)	High CD4/8 expression (n = 150)
High CD40 expression (*n* = 114)	48 (42%)	40 (35%)	59 (52%)
Low/moderate CD40 expression (*n* = 400)	59 (15%)	49 (12%)	91 (23%)
*P* value	** < 0.001**	** < 0.001**	** < 0.001**

Bold values indicate statistical significance (*P* value ≤ 0.05)

^a^Definition of RNA expression is high = 75th–100th percentile RNA rank, moderate = 25th–74th, and low = 0–24th percentile rank value score

### Co-expression of CD40 with CD28 and GITR marks an immunologically hot tumor microenvironment (n = 514)

To investigate the association between high expression of CD40 and the T-cell costimulatory molecules CD28 or GITR in an "immunologically hot" tumor microenvironment, we performed a univariable analysis stratified by the tumor's CD4/CD8 expression status (Supplementary Table S6). We found that the association of CD28 RNA expression with high CD40 expression was dependent on the CD4/CD8 context. A significant association was observed only in the patient group with high CD4/CD8 expression (OR = 2.0, 95% CI 1.02–3.9; *P* = 0.043). In contrast, the association did not reach statistical significance in patients with low/moderate CD4/CD8 expression (*P* = 0.088).

We found that high GITR RNA expression was significantly associated with high CD40 RNA expression in both patient subgroups. The association was most pronounced in patients with high CD4/CD8 expression, where high GITR levels were linked to a threefold increase in the odds of high CD40 expression (OR = 3.0, 95% CI 1.4–6.3; *P* = 0.003). This relationship remained significant, though to a lesser degree, in patients with low/moderate CD4/CD8 expression (OR = 2.2, 95% CI 1.1–4.3; *P* = 0.027).

### *CCND1 *alterations correlated with high CD40 while *APC* alterations correlated with intermediate/low CD40 RNA levels

**Fig. 6:**
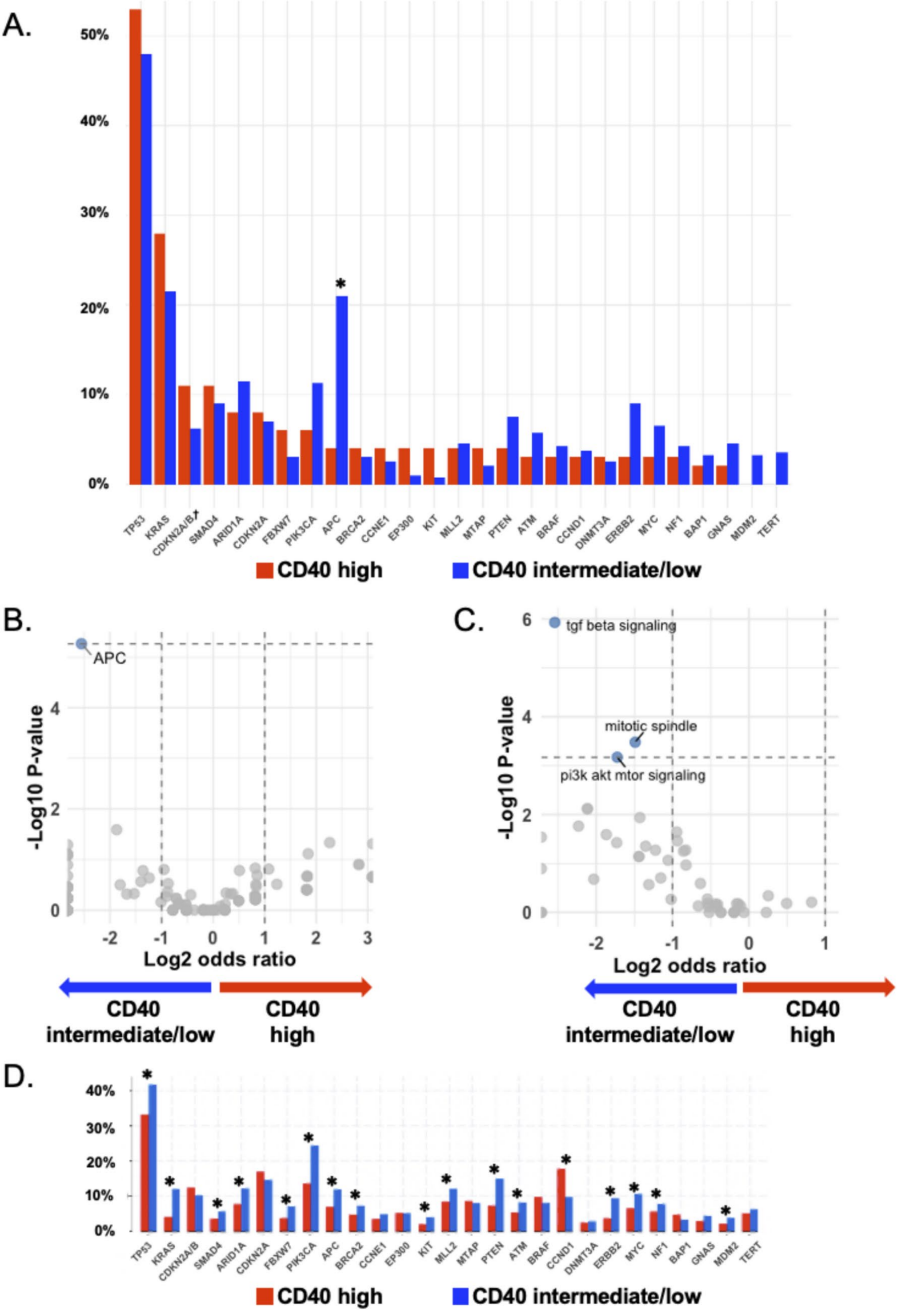
Genomic alteration frequency in patients with high (≥ 75th percentile RNA rank) versus intermediate/low (< 75th percentile RNA rank) CD40 expression. **A** The frequency of gene alterations was evaluated in patients with high (n = 114) and intermediate/low (n = 400) CD40 RNA expression from the UCSD cohort (N = 514). Each gene’s alteration frequency was compared using Fisher’s exact test with Bonferroni correction for multiple comparisons; *P* < 0.00185 (= 0.05/27) was considered statistically significant. The graph depicts the top 20 most frequently altered genes in each group, totaling 27 genes for comparison between the high and intermediate/low CD40 expression groups. Definition of RNA expression: High = 75–100th percentile RNA rank: moderate = 25–74th; and low = 0–24th percentile rank value score (see Methods). **B** Volcano plot of gene-level enrichment analysis in the UCSD cohort. The x-axis displays the log2 odds ratio, and the y-axis shows the -log10 *p* value. Each point corresponds to a single gene. Alterations in *APC* were significantly enriched in the CD40 intermediate/low expression group. **C** Volcano plot of pathway-level enrichment analysis using the 50 MSigDB Hallmark gene sets in the UCSD cohort. The TGF beta signaling, mitotic spindle, and PI3K/AKT/mTOR signaling pathways were significantly enriched in the CD40 intermediate/low group. **D** Gene alteration frequencies were similarly assessed in patients with high (n = 1740) and intermediate/low (n = 5220) CD40 RNA expression from the TCGA Pan-Cancer Atlas Studies (N = 6960). Note that CD40 expression data were unavailable for 3,993 of the overall 10,953 patients. Fisher’s exact test with Bonferroni correction for multiple comparisons was again applied. * Frequency was statistically higher in either the high-CD40 or intermediate/low-CD40 group. After Bonferroni correction, *APC* alterations were associated with intermediate/low CD40 expression in both UCSD and TCGA Pan-Cancer Atlas cohorts. † Mutations exclusively in CDKN2A were categorized as CDKN2A. CDKN2A/B homozygous deletions are shown separately under CDKN2A/B. Abbreviations: APC, adenomatous polyposis coli; CD40, cluster of differentiation 40; CDKN2A, cyclin-dependent kinase inhibitor 2A; CDKN2B, cyclin-dependent kinase inhibitor 2B; RNA, ribonucleic acid; TCGA, The Cancer Genome Atlas; UCSD, University of California, San Diego

We compared the frequency of genomic alterations between high- and intermediate/low-CD40 expression groups to characterize the gene alteration landscape (Fig. 6A). In the high-CD40 group, alterations in *TP5*3 (53%), *KRAS* (28%), *CDKN2A/B* (18%), *SMAD4* (11%), and *ARID1A* (8%) were frequently observed. In the intermediate/low-CD40 group, *TP53* (48%), *KRAS* (22%), *APC* (21%), *CDKN2A/B* (13%), and *ARID1A* (12%) were commonly altered.

When comparing alteration frequencies between these two groups, *APC* alterations were significantly more frequent in the non-high (intermediate/low) group than in the high group (after Bonferroni adjustment for multiple comparisons) (Fig. 6A). Gene-level enrichment analysis further confirmed that *APC* alterations were significantly enriched in the CD40 intermediate/low expression group (Fig. 6B). Furthermore, pathway-level enrichment analysis revealed that the TGF beta signaling, mitotic spindle, and PI3K/AKT/mTOR signaling pathways were also significantly enriched in the CD40 intermediate/low group (Fig. 6C).

The association between CD40 and *APC* was also evaluated using the TCGA Pan-Cancer Atlas cohort, confirming that *APC* alterations were significantly more frequent in the non-high CD40 group than in the high CD40 group. In contrast, *CCND1* alterations were significantly more frequent in the high vs. non-high CD40 in the TCGA cohort (Fig. 6D).

### High CD40 RNA levels are not a prognostic factor for survival in patients who never received immunotherapy

**Fig. 7:**
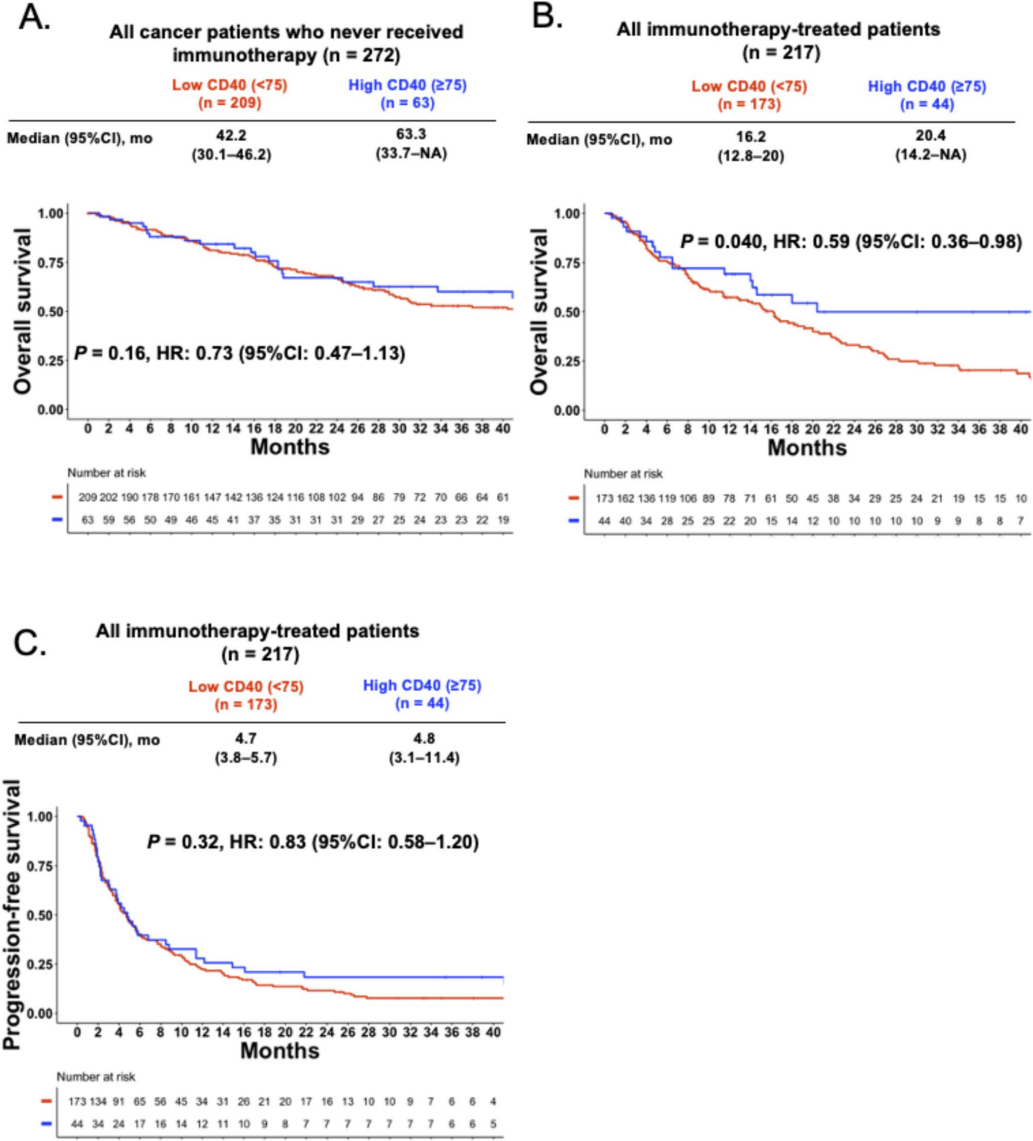
Kaplan–Meier curves for overall survival (non-immunotherapy cohort) and progression-free/overall survival (immunotherapy cohort), stratified by CD40 RNA expression. **A** Overall survival (OS) for all cancer patients who never received immunotherapy (n = 272), measured from the time of metastatic/advanced disease. The Kaplan–Meier curve shows OS from the date of metastatic/locally advanced disease to the last follow-up or death (survival data were unavailable for 25 of the 514 patients). **B** Overall survival (OS) for immunotherapy-treated patients (n = 217). The Kaplan–Meier curve shows PFS from the date of immunotherapy initiation to the earliest disease progression (clinical or radiological) or death from any cause. **C** Progression-free survival (PFS) for immunotherapy-treated patients (n = 217). The Kaplan–Meier curve shows OS for immunotherapy-treated patients from the date of immunotherapy initiation to the last follow-up or death. Definition of RNA expression: high = 75–100th, moderate = 25–74th, and low = 0–24th percentile rank value score (see Methods). Abbreviations: OS, overall survival; PFS, progression-free survival

Survival outcomes were evaluable for 489 patients: 272 never received immunotherapy, and 217 were treated with an immunotherapy-based regimen (Supplementary Fig. S1). Among those who never received immunotherapy, the median OS from advanced/metastatic disease was 63.3 months (95% CI, 33.7–Not available [NA]) in the high-CD40 group and 42.2 months (95% CI, 30.1–46.2) in the low-CD40 group (*P* = 0.16; Fig. 7A).

### High CD40 RNA levels correlated with longer OS after ICI treatment in univariable, but not in multivariable analysis

In patients treated with ICI (n = 217), OS from the start of ICI was significantly longer in high CD40 group (≥ 75th percentile RNA rank) than low/medium CD40 group (< 75th percentile RNA rank) (median, 20.4 vs. 16.2 months; HR, 0.59; 95% CI 0.36–0.98; *P* = 0.040; Fig. 7B). However, significance did not hold as an independent factor for survival in multivariable analysis (HR 0.83, 95% CI 0.47–1.36, *P* = 0.41; Supplemental Table S7). The median PFS of ICI-treated patients was 4.8 months (95% CI, 3.1–11.4) for the high-CD40 group (n = 44) and 4.7 months (95% CI, 3.8–5.7) for the low-CD40 group (*P* = 0.32; Fig. 7C).

To further validate these findings and explore a potential dose–response relationship, we also performed a univariable Cox regression analysis treating CD40 RNA expression as a continuous variable. This analysis confirmed that in the ICI-treated cohort, higher CD40 expression was significantly associated with longer OS (HR 0.991, 95% CI 0.985–0.997; *P* = 0.003) and showed a trend toward improved PFS (HR 0.995, 95% CI 0.990–1.000; *P* = 0.052). Consistent with our primary analysis, no significant association was observed for OS among those who never received immunotherapy (HR 0.997, 95% CI 0.991–1.003; *P* = 0.36) (Supplemental Table S8).

## Discussion

Immune signals via CD40 are co-stimulatory in nature [21]. Yet, CD40 agonists have shown, at best, modest responses in clinical trials (Supplemental Table S1). The current study aimed to explore transcriptomic expression patterns and clinical correlates relevant to CD40.

High CD40 expression was observed across multiple solid malignancies, especially liver/bile duct, pancreatic, and ovarian cancers. In ≥ 40% of these tumors, CD40 transcript expression was markedly elevated. The high percentage of tumor tissue expressing CD40 in pancreatic and ovarian cancers aligns with previous research [22–24]. Moreover, we propose a conceivable promising strategy: identifying and targeting patients whose tumors exhibit high CD40 expression alongside low-to-moderate CD40L expression [9, 25], though we acknowledge this hypothesis requires prospective validation. Notably, this biologically plausible pattern was detected in 33% of ovarian cancers and 24% of pancreatic cancers, indicating that these tumors may be especially amenable to exogenous CD40 stimulation. While direct evidence linking this specific biomarker to CD40 agonist response is not yet available, recent clinical data provide indirect support. For instance, in the phase 2 PRINCE trial for metastatic pancreatic cancer, survival benefit with the CD40 agonist sotigalimab correlated with baseline immune signatures of an active APC compartment and robust CD4 + T cell infiltration [26]. This suggests that a pre-existing, immune-primed microenvironment is essential for therapeutic benefit. Furthermore, in a trial for recurrent ovarian cancer, a high CD40 gene signature was a key feature in patients achieving durable clinical benefit from immunotherapy [27], linking high CD40 expression to an immunologically “hot” state permissive for response. Future clinical trials should be designed to prospectively test the predictive value of the CD40/CD40L expression profile to enrich for patients most likely to respond to this therapeutic approach.

We found that elevated CD40 expression correlates strongly with CD28 and GITR. CD28 is crucial for T-cell priming [28], whereas GITR, a member of the TNF receptor superfamily, can enhance antitumor T-cell responses [29]. Several CD28 or GITR agonists are currently under development [29–31], underscoring the need for further research to validate the potential of combination strategies targeting CD28 or GITR. Moreover, the functions of these receptors might be synergistic and non-redundant. CD28 is crucial for providing the initial "signal 2" for T-cell priming, whereas CD40 activation on APCs is essential for "licensing" them to sustain and amplify the subsequent T-cell response [20]. GITR, a member of the TNF receptor superfamily like CD40, further enhances antitumor immunity by co-stimulating effector T-cells and can mitigate suppression by regulatory T-cells. Underscoring this synergy, preclinical studies have shown that dual agonism of CD40 and GITR can produce additive antitumor effects, suggesting the potential of combination strategies that target multiple co-stimulatory pathways [32]. Furthermore, we demonstrated that the significant association between the high expression of CD40 and the T-cell costimulatory molecules CD28 or GITR was most pronounced within the context of an "immunologically hot" tumor microenvironment, defined here by high CD4/CD8 RNA expression. This finding suggests a coordinated, functional interplay between antigen-presenting cells (expressing CD40) and T-cells (expressing CD28 and GITR) that is characteristic of an active anti-tumor immune response. In parallel, leveraging immunomic profiling for patient selection may optimize therapeutic outcomes.

The heatmap revealed distinct expression patterns for immune checkpoints targeted by FDA-approved ICIs (PD-1, PD-L1, PD-L2, CTLA-4, and LAG-3). These expression was enriched in the high-CD40 group. To verify the biological specificity of this association, a differential gene expression analysis was performed between the high-CD40 group and the combined low/intermediate-CD40 groups. This analysis confirmed that while key immune checkpoint transcripts were enriched in the high-CD40 group, non-immune-related transcripts were not. These findings suggest that the co-expression of these checkpoints constitutes a specific immune signature rather than a general increase in transcriptional activity. While this relationship between CD40 and immune checkpoints was observed in the univariable analysis, it did not remain significant in our multivariable model. These findings collectively suggest a broader pro-immunogenic milieu in high-CD40 tumors, which may be harnessed through rationally designed combination therapies. While a CD40 agonist can enhance T-cell priming and activation via APCs, its efficacy may be constrained by these dominant inhibitory signals. Therefore, rationally designed combination therapies—using a CD40 agonist to stimulate the immune response while an agent like an anti-PD-1 or anti-LAG-3 antibody releases the brakes on exhausted T-cells—may be necessary to unlock the full therapeutic potential of this pathway. Previous immune correlative studies of CD40 agonists noted marked differences in circulating dendritic cells, B cells, and CD4⁺ T-cells between patients with OS exceeding 1 year versus those with OS under 1 year [33]. These observations support the guiding hypothesis that CD40 agonism may facilitate the conversion of “cold” (non-inflamed) tumors into “hot” (inflamed) tumors by enhancing APC function, T-cell priming, and immune infiltration [34]. It is important to emphasize that our study, being correlational, does not directly test this mechanistic hypothesis, which requires dedicated functional validation. Nevertheless, a phase II trial evaluating nivolumab combined with the CD40 agonist sotigalimab in patients with melanoma resistant to prior immune checkpoint inhibitors demonstrated a modest 15% overall response rate [35]. Taken together, these observations suggest that further stratification of patients based on CD40 levels and checkpoint expression may help identify individuals most likely to benefit from CD40-agonistic therapies.

From a genomic perspective, *APC* alterations occurred less frequently in the high CD40 group, a finding corroborated by external datasets. Hyperactive WNT/β-catenin signaling, often driven by *APC* loss, is a well-established pan-cancer mechanism for fostering an immune-excluded microenvironment by preventing T-cell infiltration [36, 37], providing a mechanistic basis for the observed low CD40 expression. This aligns with pathway analysis showing an enrichment of TGF-beta signaling pathway, which intersects with WNT/β-catenin signaling [38], in CD40-low tumors. Therefore, certain molecular profiles—particularly those involving hyperactive WNT signaling—may be less prevalent in tumors that rely on CD40-mediated immunomodulation. Interestingly, *CCND1* alterations, which upregulate CDK4/6 expression, were found to be significantly more frequent in tumors with high CD40 expression in the TCGA cohort. While this association did not reach statistical significance in our institutional cohort, the signal in the larger dataset suggests a complex interplay between the cell cycle and immunity. While accelerated cell cycle progression may increase genomic instability and tumor antigenicity, the same pathway has also been linked to mechanisms that suppress innate immunity, creating a state of both immune activation and evasion [39]. The functional significance of these associations for tumor immunogenicity warrants further investigation.

In our cohort of ICI-naïve patients, high CD40 expression was not an independent prognostic factor for survival, indicating that it may not universally predict clinical outcomes in the absence of immunotherapy. This finding was further strengthened when CD40 expression was treated as a continuous variable, which demonstrated a significant dose–response relationship between higher CD40 levels and improved OS. However, among patients who did receive ICIs, high CD40 expression correlated with longer survival in univariable but not in multivariable analysis. This suggests that CD40 expression is likely not an independent predictive factor for ICI efficacy but rather a component of a broader pro-immunogenic signature. We demonstrated that high CD40 expression is associated with increased CD4 and CD8 T-cell gene signatures. These findings indicate that a robust T-cell presence more frequently characterizes tumors with high CD40 expression. Previous integrative multi-omics analyses have reported elevated CD40 levels in durable responders with ovarian cancer treated with ICI-based combination [27]. Our univariable signal justifies further prospective studies to determine whether CD40 expression can help identify patients most likely to respond to immunotherapeutic strategies.

Standardization and reproducibility are critical for the clinical translation of our proposed biomarker. Using a percentile-ranking system against a large, fixed reference cohort is a robust method to overcome inter-assay variability. For prospective validation, clinical trials must use the same validated RNA sequencing assay (e.g., Oncomine Immune Response Research Assay) and locked reference population in a CLIA/CAP-certified lab. This approach ensures consistent classification of a patient’s CD40 expression as 'high,' 'moderate,' or 'low,' providing a clear and actionable framework for patient selection.

This study has several limitations. First, as a retrospective analysis, it is susceptible to unmeasured confounding. Second, the use of bulk RNA sequencing precluded determining whether tumor or immune cells are the principal contributors to CD40-related signaling. In our bulk analysis, high CD4/CD8 expression was observed in 52% of tumors with high CD40 expression, suggesting that CD40 was expressed not only in T cells but also in tumor cells within the tumor microenvironment. Future studies could leverage single-cell RNA sequencing to resolve the cellular heterogeneity of gene expression, or multiplex immunohistochemistry to visualize the spatial distribution of key protein markers. A third limitation relates to the potential impact of both temporal and spatial heterogeneity on our findings. Our analysis was based on a single, earliest-available tumor biopsy for each patient. The tumor immune microenvironment is dynamic and can be significantly altered by disease progression and intervening therapies. Therefore, the immune profile captured in an earlier biopsy may not fully represent the tumor contexture, which could lead to misclassification, where a tumor initially low in CD40 expression might evolve to become a high-expressing tumor at a later stage, or vice versa. Consequently, while our findings provide a valuable landscape of CD40 expression, the clinical application of CD40 as a predictive biomarker may require contemporary biopsies taken closer to the time of treatment to guide patient selection accurately. Fourth, it is critical to underscore that the associations identified in this study are correlational. Prospective trials incorporating functional assays are essential to validate these findings, establish causal relationships, and ultimately determine the clinical utility of using CD40 expression as a predictive biomarker for CD40-targeted therapies. Fifth, our study did not address the safety of CD40 agonist therapy, a limitation considering risks like cytokine release syndrome and hepatotoxicity, as our retrospective dataset lacked the toxicity data to correlate with transcriptomic signatures. Identifying patients with a favorable risk–benefit profile is essential. Future prospective trials should therefore collect both transcriptomic and safety data to see if immune signatures can predict adverse events, which would help optimize the therapeutic window for these potent immunotherapies. Sixth, the heterogeneity of our pan-cancer cohort and the modest sample sizes for individual tumor types, such as the 55 pancreatic and 43 ovarian cancer cases, limited the statistical power for more granular analyses. Future studies focusing on specific histologic subtypes are needed to enhance the clinical and translational relevance of these findings. Seventh, although this work suggests the high-CD40/low-moderate-CD40L profile as a potential biomarker for CD40 agonist therapy, the clinical utility of this pattern could not be assessed due to small subgroup sizes and requires validation in further research. Finally, future research should integrate CD40 expression into multidimensional models to refine patient selection. For example, in renal cell carcinoma, machine learning can analyze transcriptomic data alongside genomic and metabolic resistance markers like mTOR and mitochondrial DNA alterations [40]. Embedding CD40 signatures in these models can reveal links to specific molecular vulnerabilities, uncovering novel biomarkers to enable more precise, tailored combination immunotherapies.

In conclusion, our findings underscore the potential value of incorporating transcriptomic analyses into immunotherapeutic strategies for targeting CD40. Coupled with mounting evidence regarding the synergy between CD40 agonists and other immunotherapies, these results advocate for further exploration of CD40-based interventions tailored to specific tumor types and unique molecular and immunologic profiles.

## Supplementary Information

Below is the link to the electronic supplementary material.

Supplementary file1 (DOCX 167 KB)

## Data Availability

The data that support the findings of this study are available from the corresponding author upon reasonable request.
