# Supplementary material for: CD40 transcriptomic expression patterns across malignancies: implications for clinical trials of CD40 agonists
Source: Cancer Immunol Immunother. 2025 Nov 3;74(12):359. doi: 10.1007/s00262-025-04197-8 (PMC12583269; doi:10.1007/s00262-025-04197-8)
Supplement: Supplementary file 1 — Supplementary file1 (DOCX 167 KB) [file 262_2025_4197_MOESM1_ESM.docx]

**Supplemental Figure 1. Patient flow diagram**


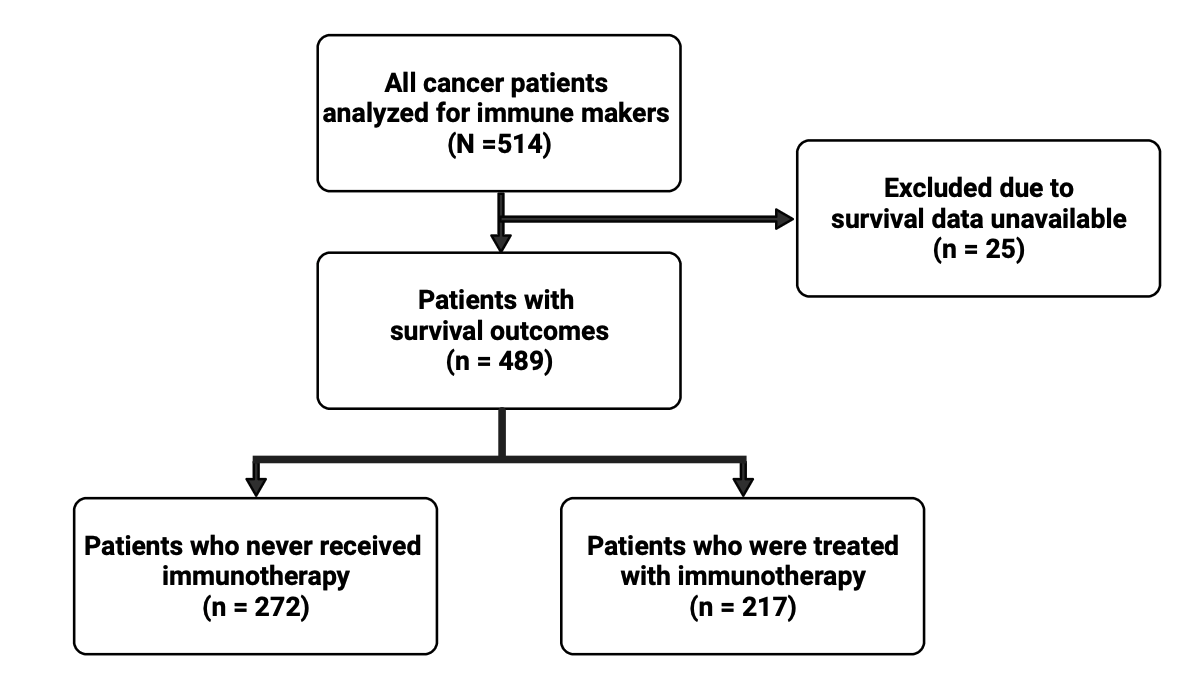


**Supplemental Table S1. Examples of clinical trials targeting CD40**

| **Agent** | **Combination**  **under clinical**  **evaluation** | **Additional**  **biological**  **agent** | **Condition** | **Phase (Status)** | **Status of**  **CD40 expression required for enrollment according to clinical trial.gov** | **Trial number** | **Objective**  **response**  **rate (n = total patients treated)** | **Reference** |
| --- | --- | --- | --- | --- | --- | --- | --- | --- |
| **CD40 agonist antibody** | | | | | | | | |
| Sotigalimab (APX005M) |  |  | Solid tumors | I (completed) | None | NCT02482168 |  |  |
|  |  |  | Sarcoma | II (recruiting) | None | NCT03719430 | 16% (n=31) | [1] |
|  |  |  | Melanoma | II (completed) | None | NCT04337931 | 15% (n=33) | [2] |
|  |  |  | Pediatric CNS tumors | I (not recruiting) | None | NCT03389802 |  |  |
|  | Nivolumab  (anti-PD-1 antibody) | Nab-Paclitaxel  , Gemcitabine | Pancreatic adenocarcinoma | I and II (completed) | None | NCT03214250 | 58% (n=14) | [3] |
|  | Nivolumab  (anti-PD-1 antibody) |  | Solid tumors | I and II (completed) | None | NCT03123783 | 18% (n=38) | [4] |
|  | Pembrolizumab (anti-PD-1 antibody) |  | Melanoma | I and II (not recruiting) | None | NCT02706353 | 55% (n=22) | [5] |
|  | mFOLFOX |  | Rectal cancer | II (not recruiting) | None | NCT04130854 |  |  |
|  | Ipilimumab (anti-CTLA-4 antibody), Nivolumab  (anti-PD-1 antibody) | NEO-PV-01 (cancer vaccine) | Melanoma | I (terminated) | None | NCT03597282 |  |  |
| CDX-1140 |  |  | Solid tumors | I (completed) | None | NCT03329950 | 2% (n=92) | [6] |
|  | Pembrolizumab (anti-PD-1) |  | Solid tumors | I (completed) | None | NCT03329950 | 10% (n=10) | [7] |
|  | Pembrolizumab (anti-PD-1) | Bevacizumab (anti-VEGF antibody) | Ovarian cancer | II (not recruiting) | None | NCT05231122 |  |  |
|  | CDX-301 (FLT3 ligand) | Chemotherapy | Triple negative breast cancer | I (recruiting) | None | NCT05029999 |  |  |
|  | CDX-301 (FLT3 ligand) | Stereotactic Radiotherapy | NSCLC | I and II (terminated) | None | NCT04491084 |  |  |
|  | Melanoma vaccine against neoantigen and shared antigens | Poly-ICLC (toll-like receptor 3 agonist) | Melanoma | I and II (not recruiting) | None | NCT04364230 |  |  |
|  | Pembrolizumab (anti-PD-1) | Capecitabine, Oxaliplatin | Biliary tract carcinoma | I and II (not yet recruiting) | None | NCT05849480 |  |  |
|  | Odetiglucan (soluble beta-glucan) |  | Pancreatic ductal adenocarcinoma | I (not recruiting) | None | NCT05484011 |  |  |
| Lucatumumab  (HCD122) |  |  | Multiple myeloma | I (completed) | None | NCT00231166 | 4% (n=28) | [8] |
|  | Bendamustine |  | Follicular lymphoma | I (completed) | Yes | NCT01275209 |  |  |
|  |  |  | Chronic lymphocytic leukemia | I (terminated) | None | NCT00108108 | 4% (n=26) | [9] |
|  |  |  | Non-hodgkin's or Hodgkin’s lymphoma | I and II (completed) | None | NCT00670592 | 19% (n=74**,** Non-hodgkin's lymphoma**),**  14% (n=37, Hodgkin’s lymphoma) | [10] |
| LVGN7409 |  |  | Solid tumors | I (recruiting) | None | NCT05152212 | 0% (n=9) | [11] |
|  |  |  |  |  |  | NCT04635995 |  |  |
|  | LVGN3616 (Anti-PD-1 antibody |  | Solid tumors | I (recruiting) | None | NCT04635995 |  |  |
|  | LVGN6051 (CD137 agonist antibody) |  | Solid tumors | I (recruiting) | None | NCT04635995 |  |  |
| Mitazalimab  (ADC-1013,  JNJ-64457107) |  |  | Solid tumors | I (completed) | None | NCT02379741 |  |  |
|  |  |  | Solid tumors | I (completed) | None | NCT02829099 | 1% (n=95) | [12] |
|  | MesoPher (Dendritic cells loaded with allogeneic tumor lysate ) |  | Pancreatic tumor | I (completed) | None | NCT05650918 |  |  |
| Chi Lob 7/4 |  |  | Solid tumors | I (completed) | None | NCT01561911 |  |  |
| 2141-V11 |  |  | Bladder cancer | I (recruiting) | None | NCT05126472 |  |  |
|  | D2C7-IT (D2C7 immunotoxin) |  | Malignant glioma | I (recruiting) | None | NCT04547777 |  |  |
| Dacetuzumab (SEA‑CD40, SGN‑40) |  |  | Solid tumors | I (terminated) | None | NCT02376699 | 3% (n=67) | [13] |
|  |  |  | Non-Hodgkin's lymphoma | I (completed) | None | NCT00103779 | 12% (n=50) | [14] |
|  |  |  | Chronic lymphocytic leukemia | I (completed) | None | NCT00283101 |  |  |
|  |  |  | DLBCL | II (completed) | None | NCT00435916 | 9% (n=46) | [15] |
|  |  |  | Multiple myeloma | I (completed) | None | NCT00079716 | 0% (n=44) | [16] |
|  | Pembrolizumab (anti-PD-1) | Carboplatin, Pemetrexed | Solid tumors | II (not recruiting) | None | NCT04993677 |  |  |
|  | Bortezomib (selective inhibitor of the 26S proteasome) |  | Multiple myeloma | I (completed) | None | NCT00664898 |  |  |
|  | Rituximab (anti-CD20 antibody) |  | Follicular and Marginal zone B-cell non-hodgkin's lymphoma | I (completed) | None | NCT00556699 |  |  |
|  | Rituximab (anti-CD20 antibody) | Etoposide, Carboplatin, Ifosfamide | DLBCL | II (terminated) | None | NCT00529503 | 66% (n=51) | [17] |
|  | Rituximab (anti-CD20 antibody) | Chemotherapy | DLBCL | I (completed) | None | NCT00655837 |  |  |
|  | Lenalidomide | Dexamethasone | Multiple myeloma | I (completed) | None | NCT00525447 |  |  |
| Selicrelumab | Atezolizumab (anti-PD-L1 antibody) |  | Solid tumors | I (completed) | None | NCT02304393 | 9% (n=80) | [18] |
|  | Atezolizumab (anti-PD-L1 antibody) |  | B cell lymphoma | I (terminated) | None | NCT03892525 |  |  |
|  | Vanucizumab (Atezolizumab (anti-Ang-2/VEGF bispecific antibody antibody)) |  | Solid tumors | I (completed) | None | NCT02665416 |  |  |
|  | Bevacizumab (anti-VEGF antibody) |  |  |  |  |  |  |  |
| CP-870,893  (RO7009789) |  |  | Solid tumors | I (completed) | None | NCT02157831 | 14% (n=29) | [19] |
|  | Tremelimumab (anti-CTLA4 antibody) |  | Melanoma | I (completed) | None | NCT01103635 | 18% (n=22) | [20] |
|  | Gemcitabine |  | Pancreatic cancer | I (completed) | None | NCT00711191 | 18% (n=22) | [21] |
| **Bispecific antibody, Bifunctional protein** | | | | | | | | |
| ABBV-428  (CD40 x mesothelin bispecific antibody) |  |  | Solid tumors | I (completed) | None | NCT02955251 | 0% (n=53) | [22] |
| RO7300490  (CD40 x FAP bispecific antibody) |  |  | Solid tumors | I (recruiting) | None | NCT04857138 | 0% (n=26) | [23] |
| MP0317 (designed ankyrin repeat protein targeting FAP and CD40) |  |  | Solid tumors | I (recruiting) | None | NCT05098405 | 3% (n=36) | [24] |
| GEN1042  (CD40 x 4–1BB bispecific antibody) |  |  | Solid tumors | I and II (recruiting) | None | NCT04083599 | 4% (n=51) | [25] |
|  | Pembrolizumab (anti-PD-1) | Chemotherapy | Solid tumors | I and II (recruiting) | None | NCT04083599 |  |  |
| **Adenoviral vector** | | | | | | | | |
| NG-350A ( Oncolytic adenoviral vector which expresses an anti-CD40 antibody) |  |  | Solid tumors | I (completed) | None | NCT03852511 | 0% (n=28) | [26] |
|  | Pembrolizumab (anti-PD-1) |  | Epithelial tumors | I (recruiting) | None | NCT05165433 |  |  |
|  | Ipilimumab (anti-CTLA-4 antibody) | Gemcitabine or Nab-paclitaxel | Pancreatic cancer | I (not recruiting) | None | NCT04787991 |  |  |

**Abbreviations**: CNS, central nervous system; DLBCL, diffuse large B-cell lymphoma; FAP, fibroblast activation protein; NSCLC, non-small cell lung cancer.

Last searched on 11/24/2023 at ClinicalTrials.gov and Cochrane Central Register of Controlled Trials.

**References for Supplemental Table S1**

1. Bose S (Sam), Ge L, Lee SM, et al (2023) A phase II trial with safety lead-in to evaluate the addition of sotigalimab, a CD40 agonistic monoclonal antibody, to standard-of-care doxorubicin for the treatment of advanced sarcoma. JCO 41:11565–11565. https://doi.org/10.1200/JCO.2023.41.16_suppl.11565

2. Weiss SA, Sznol M, Shaheen M, et al (2023) A Phase II Trial of the CD40 Agonist Sotigalimab (APX005M) in Combination with Nivolumab in Subjects with Metastatic Melanoma with Disease Progression on Anti-PD-1. Clin Cancer Res CCR-23-0475. https://doi.org/10.1158/1078-0432.CCR-23-0475

3. O’Hara MH, O’Reilly EM, Varadhachary G, et al (2021) CD40 agonistic monoclonal antibody APX005M (sotigalimab) and chemotherapy, with or without nivolumab, for the treatment of metastatic pancreatic adenocarcinoma: an open-label, multicentre, phase 1b study. Lancet Oncol 22:118–131. https://doi.org/10.1016/S1470-2045(20)30532-5

4. Weiss S, Sznol M, Shaheen M, et al (2021) 389 Phase II of CD40 agonistic antibody sotigalimab (APX005M) in combination with nivolumab in subjects with metastatic melanoma with confirmed disease progression on anti-PD-1 therapy. J Immunother Cancer 9:. https://doi.org/10.1136/jitc-2021-SITC2021.389

5. Bentebibel S-E, Johnson D, Pazdrak B, et al (2022) 782 Intratumoral sotigalimab with pembrolizumab activates antigen-presenting cells and induces local and distant anti-tumor responses in first-line metastatic melanoma: results of a phase I/II study. J Immunother Cancer 10:. https://doi.org/10.1136/jitc-2022-SITC2022.0782

6. Sanborn R, Hauke R, Gabrail N, et al (2020) 405 CDX1140–01, a phase 1 dose-escalation/expansion study of CDX-1140 alone (Part 1) and in combination with CDX-301 (Part 2) or pembrolizumab (Part 3). J Immunother Cancer 8:. https://doi.org/10.1136/jitc-2020-SITC2020.0405

7. Sanborn R, Gabrail N, Carneiro B, et al (2022) 596 Results from a phase 1 study of CDX-1140, a fully human anti-CD40 agonist monoclonal antibody (mAb), in combination with pembrolizumab. J Immunother Cancer 10:. https://doi.org/10.1136/jitc-2022-SITC2022.0596

8. Bensinger W, Maziarz RT, Jagannath S, et al (2012) A phase 1 study of lucatumumab, a fully human anti-CD40 antagonist monoclonal antibody administered intravenously to patients with relapsed or refractory multiple myeloma. British Journal of Haematology 159:58–66. https://doi.org/10.1111/j.1365-2141.2012.09251.x

9. Byrd JC, Kipps TJ, Flinn IW, et al (2012) Phase I study of the anti-CD40 humanized monoclonal antibody lucatumumab (HCD122) in relapsed chronic lymphocytic leukemia. Leuk Lymphoma 53:2136–2142. https://doi.org/10.3109/10428194.2012.681655

10. Fanale M, Assouline S, Kuruvilla J, et al (2014) Phase IA/II, multicentre, open-label study of the CD40 antagonistic monoclonal antibody lucatumumab in adult patients with advanced non-Hodgkin or Hodgkin lymphoma. Br J Haematol 164:258–265. https://doi.org/10.1111/bjh.12630

11. Fu S, Vandross AL, Hsu YH, et al (2022) Early safety and efficacy from a phase I open-label clinical study of LVGN7409 (CD40 agonist antibody) in patients with advanced or metastatic malignancies. JCO 40:e14501–e14501. https://doi.org/10.1200/JCO.2022.40.16_suppl.e14501

12. Moreno V, Perets R, Peretz-Yablonski T, et al (2023) A phase 1 study of intravenous mitazalimab, a CD40 agonistic monoclonal antibody, in patients with advanced solid tumors. Invest New Drugs 41:93–104. https://doi.org/10.1007/s10637-022-01319-2

13. Coveler AL, Smith DC, Phillips T, et al (2023) Phase 1 dose-escalation study of SEA-CD40: a non-fucosylated CD40 agonist, in advanced solid tumors and lymphomas. J Immunother Cancer 11:e005584. https://doi.org/10.1136/jitc-2022-005584

14. Advani R, Forero-Torres A, Furman RR, et al (2009) Phase I study of the humanized anti-CD40 monoclonal antibody dacetuzumab in refractory or recurrent non-Hodgkin’s lymphoma. J Clin Oncol 27:4371–4377. https://doi.org/10.1200/JCO.2008.21.3017

15. de Vos S, Forero-Torres A, Ansell SM, et al (2014) A phase II study of dacetuzumab (SGN-40) in patients with relapsed diffuse large B-cell lymphoma (DLBCL) and correlative analyses of patient-specific factors. Journal of Hematology & Oncology 7:44. https://doi.org/10.1186/1756-8722-7-44

16. Hussein M, Berenson JR, Niesvizky R, et al (2010) A phase I multidose study of dacetuzumab (SGN-40; humanized anti-CD40 monoclonal antibody) in patients with multiple myeloma. Haematologica 95:845–848. https://doi.org/10.3324/haematol.2009.008003

17. Fayad L, Ansell SM, Advani R, et al (2015) Dacetuzumab plus rituximab, ifosfamide, carboplatin and etoposide as salvage therapy for patients with diffuse large B-cell lymphoma relapsing after rituximab, cyclophosphamide, doxorubicin, vincristine and prednisolone: a randomized, double-blind, placebo-controlled phase 2b trial. Leukemia & Lymphoma 56:2569–2578. https://doi.org/10.3109/10428194.2015.1007504

18. Barlesi F, Lolkema M, Rohrberg KS, et al (2020) 291 Phase Ib study of selicrelumab (CD40 agonist) in combination with atezolizumab (anti-PD-L1) in patients with advanced solid tumors. J Immunother Cancer 8:. https://doi.org/10.1136/jitc-2020-SITC2020.0291

19. Vonderheide RH, Flaherty KT, Khalil M, et al (2007) Clinical Activity and Immune Modulation in Cancer Patients Treated With CP-870,893, a Novel CD40 Agonist Monoclonal Antibody. JCO 25:876–883. https://doi.org/10.1200/JCO.2006.08.3311

20. Bajor DL, Mick R, Riese MJ, et al (2018) Long-term outcomes of a phase I study of agonist CD40 antibody and CTLA-4 blockade in patients with metastatic melanoma. Oncoimmunology 7:e1468956. https://doi.org/10.1080/2162402X.2018.1468956

21. Beatty GL, Torigian DA, Chiorean EG, et al (2013) A phase I study of an agonist CD40 monoclonal antibody (CP-870,893) in combination with gemcitabine in patients with advanced pancreatic ductal adenocarcinoma. Clin Cancer Res 19:6286–6295. https://doi.org/10.1158/1078-0432.CCR-13-1320

22. Luke JJ, Barlesi F, Chung K, et al (2021) Phase I study of ABBV-428, a mesothelin-CD40 bispecific, in patients with advanced solid tumors. J Immunother Cancer 9:e002015. https://doi.org/10.1136/jitc-2020-002015

23. Melero I, Baradji ML, Spanggaard I, et al (2023) 617 A Phase I study of a tumor-targeted, fibroblast activation protein (FAP)-CD40 agonist (RO7300490) in patients with advanced solid tumors. J Immunother Cancer 11:. https://doi.org/10.1136/jitc-2023-SITC2023.0617

24. Gomez-Roca C, Steeghs N, Gort E, et al (2023) 721 Ongoing phase 1 study of MP0317, a FAP-CD40 DARPin, shows a favorable safety profile and early evidence of tumor-localized CD40 activation in patients with advanced solid tumors. J Immunother Cancer 11:. https://doi.org/10.1136/jitc-2023-SITC2023.0721

25. Johnson M, Lopez J, LoRusso P, et al (2021) 493 First-in-human phase 1/2 trial to evaluate the safety and initial clinical activity of DuoBody®-CD40×4–1BB (GEN1042) in patients with advanced solid tumors. J Immunother Cancer 9:. https://doi.org/10.1136/jitc-2021-SITC2021.493

26. Rosen LS, Camidge DR, Khalil D, et al (2022) FORTITUDE: Results of a phase 1a study of the novel transgene-armed and tumor-selective vector NG-350A with and without pembrolizumab (pembro). JCO 40:2559–2559. https://doi.org/10.1200/JCO.2022.40.16_suppl.2559

**Supplemental Table S2. Patient characteristics (N = 514)**

| **Characteristic** | **N = 514** |
| --- | --- |
| Age, median (range, years)  Tumor Biopsy Sites  Metastatic sites  Primary sites | 61 (24–93)  429 (84%)  85 (16%) |
| Gender, n (%) |  |
| Female | 310 (60%) |
| Male | 204 (40%) |
| PD-L1 IHC CPS ≥1, n (%)^a^ | 156 (30%) |
| TMB ≥10 (mutations/Megabase), n (%)^a^ | 33 (7%) |
| MSI unstable, n (%)^a^ | 15 (3%) |
| Disease, n (%) |  |
| Colorectal Cancer | 140 (27%) |
| Pancreatic Cancer | 55 (11%) |
| Breast Cancer | 49 (9.5%) |
| Ovarian Cancer | 43 (8.4%) |
| Stomach Cancer | 25 (4.9%) |
| Sarcoma | 24 (4.7%) |
| Uterine Cancer | 24 (4.7%) |
| Lung Cancer | 20 (3.9%) |
| Liver and Bile Duct Cancer | 19 (3.7%) |
| Esophageal Cancer | 17 (3.3%) |
| Neuroendocrine Tumors | 15 (2.9%) |
| Unknown Primary Cancer | 13 (2.5%) |
| Head and Neck Cancer | 12 (2.3%) |
| Small Intestine Cancer | 12 (2.3%) |
| Other^b^ | 46 (8.9%) |
| **Abbreviations**: CPS, combined positive score; IHC, immunohistochemistry; MSI, microsatellite instability; TMB, tumor mutation burden.  ^a^ Tabulation was performed only among patients with available TMB (n = 450), MSI (n = 480), and PD-L1 IHC (n = 513). | |
| ^b^ Cancers that had at least 10 patients were listed. Other includes melanoma (n = 6), cervical cancer (n = 5), bladder Cancer (n = 4), gallbladder and extrahepatic bile duct cancers (n = 4), prostate cancer (n = 4), brain and nervous system cancer (n = 3), kidney and renal pelvis cancer (n = 3), squamous cell carcinoma of the skin (n = 3), thyroid cancer (n = 3), adrenal gland cancer (n = 2), lipomatous neoplasms (n = 2), mesothelioma (n = 2), adrenal cortical carcinoma (n = 1), basal cell carcinoma of the skin (n = 1), ocular melanoma (n = 1), primary peritoneal carcinoma (n = 1), and thymic cancer (n = 1). | |

**Supplemental Table S3. Immune markers examined in this study and their function**

| **Molecule** | **Alternative Names** | **Function** | **References** |
| --- | --- | --- | --- |
| CD40 | TNFRSF5 | CD40 is expressed in cancer cells, B cells, and antigen-presenting cells. CD40 ligand is expressed in activated T cells.  Binding of the CD40 ligand to CD40 results in an immune stimulatory signal.  Stimulating this axis may lead to an anti-cancer effect. | [1] |
| 4-1BB | TNFRSF9/CD137 | CD137 is primarily expressed in activated T cells and NK cells. CD137 ligand can be expressed in cancer cells, antigen-presenting cells, or B cells.  Binding of the CD137 ligand to CD137 results in an immune stimulatory signal.  Stimulating this axis may lead to an anti-cancer effect. | [2] |
| PD-1 | CD279 | PD-L1 and PD-L2 can be expressed in cancer cells or antigen-presenting cells. PD-1 is expressed in activated T cells.  Binding of PD-L1/PD-L2 to PD-1 results in an immune inhibitory signal.  Inhibiting this axis can lead to an anti-cancer effect. | [3] |
| PD-L1 | CD274, B7-H1 |  |  |
| PD-L2 | PDCD1LG2, B7-DC |  |  |
| CTLA-4 | CD152 | CTLA-4 is expressed in regulatory T cells. CD80/CD86, a ligand for CTLA-4, is expressed in antigen-presenting cells.  Binding of CD80/CD86 to CTLA-4 leads to an immune inhibitory signal.  Inhibiting this axis can produce an anti-cancer effect. | [4] |
| LAG-3 | CD223 | LAG3 is expressed in cells including activated T cells, NK cells. MHC class II, a major ligand for LAG3, is expressed in antigen-presenting cells and activated T cells.  Binding of MHC class II to LAG3 leads to an immune inhibitory signal.  Inhibiting this axis can lead to an anti-cancer effect. | [5] |
| ICOS | CD278 | ICOS is expressed in activated T cells, NK cells. ICOS ligand is expressed in antigen-presenting cells.  Binding of ICOS ligand to ICOS results in an immune stimulatory signal.  Stimulating this axis may lead to an anti-cancer effect. | [6] |
| CD27 | TNFRSF7 | CD27 is expressed in naïve and memory T cells. CD70, a ligand for CD27, is expressed in activated T cells, B cells, and antigen-presenting cells.  Binding of CD70 to CD27 results in an immune stimulatory signal.  Stimulating this axis may lead to an anti-cancer effect. | [7] |
| CD28 |  | CD28 is expressed in activated T cells. CD80/CD86, ligands for CD28, are expressed in antigen-presenting cells.  Binding of CD80/CD86 to CD28 (instead of CTLA-4) results in an immune stimulatory signal.  Stimulating this axis may lead to an anti-cancer effect. | [8] |
| OX40 | TNFRSF4, CD134 | OX40 is expressed in activated T cells, NK cells, and neutrophils. OX40 ligand (CD134L, CD252) is expressed in antigen-presenting cells and B cells.  Binding of OX40 ligand to OX40 results in an immune stimulatory signal.  Stimulating this axis can lead to an anti-cancer effect. | [9] |
| GITR | TNFRSF18 | GITR is expressed in regulatory, naïve, and memory T cells. GITR ligand is expressed in antigen-presenting cells and B cells.  Binding of the GITR ligand to GITR results in an immune stimulatory signal.  Stimulating this axis may lead to an anti-cancer effect. | [10] |

**References for Supplemental Table S2**

1. Bullock TNJ (2022) CD40 stimulation as a molecular adjuvant for cancer vaccines and other immunotherapies. Cell Mol Immunol 19:14–22. https://doi.org/10.1038/s41423-021-00734-4

2. Etxeberria I, Glez-Vaz J, Teijeira Á, Melero I (2020) New emerging targets in cancer immunotherapy: CD137/4-1BB costimulatory axis. ESMO Open 4:e000733. https://doi.org/10.1136/esmoopen-2020-000733

3. Gong J, Chehrazi-Raffle A, Reddi S, Salgia R (2018) Development of PD-1 and PD-L1 inhibitors as a form of cancer immunotherapy: a comprehensive review of registration trials and future considerations. J Immunother Cancer 6:8. https://doi.org/10.1186/s40425-018-0316-z

4. Walker LSK, Sansom DM (2011) The emerging role of CTLA4 as a cell-extrinsic regulator of T cell responses. Nat Rev Immunol 11:852–863. https://doi.org/10.1038/nri3108

5. Maruhashi T, Sugiura D, Okazaki I, Okazaki T (2020) LAG-3: from molecular functions to clinical applications. J Immunother Cancer 8:e001014. https://doi.org/10.1136/jitc-2020-001014

6. Solinas C, Gu-Trantien C, Willard-Gallo K (2020) The rationale behind targeting the ICOS-ICOS ligand costimulatory pathway in cancer immunotherapy. ESMO Open 5:e000544. https://doi.org/10.1136/esmoopen-2019-000544

7. Starzer AM, Berghoff AS (2020) New emerging targets in cancer immunotherapy: CD27 (TNFRSF7). ESMO Open 4:e000629. https://doi.org/10.1136/esmoopen-2019-000629

8. Esensten JH, Helou YA, Chopra G, et al (2016) CD28 costimulation: from mechanism to therapy. Immunity 44:973–988. https://doi.org/10.1016/j.immuni.2016.04.020

9. Webb GJ, Hirschfield GM, Lane PJL (2016) OX40, OX40L and Autoimmunity: a Comprehensive Review. Clin Rev Allergy Immunol 50:312–332. https://doi.org/10.1007/s12016-015-8498-3

10. Buzzatti G, Dellepiane C, Del Mastro L (2020) New emerging targets in cancer immunotherapy: the role of GITR. ESMO Open 4:e000738. https://doi.org/10.1136/esmoopen-2020-000738

**Supplemental Table S4: Gene lists from RNA sequencing using the Oncomine Immune Response Research Assay**

| Gene | Target NCBI NAME | Gene function |
| --- | --- | --- |
| BCL6 | B-cell CLL/lymphoma 6 | Type II interferon signaling |
| CIITA | class II major histocompatibility complex transactivator | Type II interferon signaling |
| CX3CL1 | C-X3-C motif chemokine ligand 1 | Type II interferon signaling |
| CXCL10 | C-X-C motif chemokine ligand 10 | Type II interferon signaling |
| CXCL11 | C-X-C motif chemokine ligand 11 | Type II interferon signaling |
| CXCL13 | C-X-C motif chemokine ligand 13 | Type II interferon signaling |
| CXCL9 | C-X-C motif chemokine ligand 9 | Type II interferon signaling |
| CXCR5 | C-X-C motif chemokine receptor 5 | Type II interferon signaling |
| CYBB | cytochrome b-245 beta chain | Type II interferon signaling |
| EIF2AK2 | eukaryotic translation initiation factor 2 alpha kinase 2 | Type II interferon signaling |
| FASLG | Fas ligand | Type II interferon signaling |
| GBP1 | guanylate binding protein 1 | Type II interferon signaling |
| ICAM1 | intercellular adhesion molecule 1 | Type II interferon signaling |
| IFNB1 | interferon beta 1 | Type II interferon signaling |
| IFNG | interferon gamma | Type II interferon signaling |
| IL1B | interleukin 1 beta | Type II interferon signaling |
| IRF1 | interferon regulatory factor 1 | Type II interferon signaling |
| IRF9 | interferon regulatory factor 9 | Type II interferon signaling |
| OAS1 | 2'-5'-oligoadenylate synthetase 1 | Type II interferon signaling |
| PSMB9 | proteasome subunit beta 9 | Type II interferon signaling |
| STAT1 | signal transducer and activator of transcription 1 | Type II interferon signaling |
| TAP1 | transporter 1, ATP-binding cassette sub-family B (MDR/TAP) | Type II interferon signaling |
| TBX21 | T-box 21 | Type II interferon signaling |
| BST2 | bone marrow stromal cell antigen 2 | Type I interferon signaling |
| IFI27 | interferon alpha inducible protein 27 | Type I interferon signaling |
| IFIT1 | interferon induced protein with tetratricopeptide repeats 1 | Type I interferon signaling |
| IFIT3 | interferon induced protein with tetratricopeptide repeats 3 | Type I interferon signaling |
| IFITM1 | interferon induced transmembrane protein 1 | Type I interferon signaling |
| IFITM2 | interferon induced transmembrane protein 2 | Type I interferon signaling |
| ISG15 | ISG15 ubiquitin-like modifier | Type I interferon signaling |
| ISG20 | interferon stimulated exonuclease gene 20 | Type I interferon signaling |
| SNAI1 | snail family transcriptional repressor 1 | Tumor marker, stemness |
| SNAI2 | snail family transcriptional repressor 2 | Tumor marker, stemness |
| TWIST1 | twist family bHLH transcription factor 1 | Tumor marker, stemness |
| ZEB1 | zinc finger E-box binding homeobox 1 | Tumor marker, stemness |
| AKT1 | AKT serine/threonine kinase 1 | Tumor marker |
| BRCA1 | BRCA1 DNA repair associated | Tumor marker |
| BRCA2 | BRCA2 DNA repair associated | Tumor marker |
| CDKN2A | cyclin-dependent kinase inhibitor 2A | Tumor marker |
| EFNA4 | ephrin A4 | Tumor marker |
| EGFR | epidermal growth factor receptor | Tumor marker |
| EGR3 | early growth response 3 | Tumor marker |
| IRS1 | insulin receptor substrate 1 | Tumor marker |
| KRT5 | keratin 5 | Tumor marker |
| KRT7 | keratin 7 | Tumor marker |
| MAPK1 | mitogen-activated protein kinase 1 | Tumor marker |
| MMP2 | matrix metallopeptidase 2 | Tumor marker |
| MMP9 | matrix metallopeptidase 9 | Tumor marker |
| MYC | v-myc avian myelocytomatosis viral oncogene homolog | Tumor marker |
| NOTCH3 | notch 3 | Tumor marker |
| PGF | placental growth factor | Tumor marker |
| PTGS2 | prostaglandin-endoperoxide synthase 2 | Tumor marker |
| PTK7 | protein tyrosine kinase 7 (inactive) | Tumor marker |
| RB1 | RB transcriptional corepressor 1 | Tumor marker |
| RPS6 | ribosomal protein S6 | Tumor marker |
| TCF7 | transcription factor 7, T-cell specific, HMG-box | Tumor marker |
| TP63 | tumor protein p63 | Tumor marker |
| TRIM29 | tripartite motif containing 29 | Tumor marker |
| BAGE | B melanoma antigen | Tumor antigen |
| CTAG1B | cancer testis antigen 1B | Tumor antigen |
| CTAG2 | cancer testis antigen 2 | Tumor antigen |
| GAGE1, GAGE12I, GAGE12F | G antigen 1 | Tumor antigen |
| GAGE10 | G antigen 10 | Tumor antigen |
| GAGE12J | G antigen 12J | Tumor antigen |
| GAGE13 | G antigen 13 | Tumor antigen |
| GAGE2C, GAGE2A, GAGE2E | G antigen 2C | Tumor antigen |
| MAGEA1 | MAGE family member A1 | Tumor antigen |
| MAGEA10 | MAGE family member A10 | Tumor antigen |
| MAGEA12 | MAGE family member A12 | Tumor antigen |
| MAGEA3 | MAGE family member A3 | Tumor antigen |
| MAGEA4 | MAGE family member A4 | Tumor antigen |
| MAGEC2 | MAGE family member C2 | Tumor antigen |
| MLANA | melan-A | Tumor antigen |
| SSX2 | SSX family member 2 | Tumor antigen |
| XAGE1B | X antigen family member 1B | Tumor antigen |
| CCR7 | C-C motif chemokine receptor 7 | TCR coexpression |
| CD247 | CD247 molecule | TCR coexpression |
| CD3D | CD3d molecule | TCR coexpression |
| CD3E | CD3e molecule | TCR coexpression |
| CD3G | CD3g molecule | TCR coexpression |
| CD6 | CD6 molecule | TCR coexpression |
| CD8A | CD8a molecule | TCR coexpression |
| CD8B | CD8b molecule | TCR coexpression |
| CRTAM | cytotoxic and regulatory T-cell molecule | TCR coexpression |
| GPR18 | G protein-coupled receptor 18 | TCR coexpression |
| GRAP2 | GRB2-related adaptor protein 2 | TCR coexpression |
| IKZF3 | IKAROS family zinc finger 3 | TCR coexpression |
| IL2RB | interleukin 2 receptor subunit beta | TCR coexpression |
| IL7R | interleukin 7 receptor | TCR coexpression |
| ITK | IL2 inducible T-cell kinase | TCR coexpression |
| LAMP3 | lysosomal associated membrane protein 3 | TCR coexpression |
| LCK | LCK proto-oncogene, Src family tyrosine kinase | TCR coexpression |
| PTPRCAP | protein tyrosine phosphatase receptor type C associated protein | TCR coexpression |
| TIGIT | T cell immunoreceptor with Ig and ITIM domains | TCR coexpression |
| KLF2 | Kruppel like factor 2 | T cell regulation, trafficking |
| EBI3 | Epstein-Barr virus induced 3 | T cell regulation |
| FOXP3 | forkhead box P3 | T cell regulation |
| ID2 | inhibitor of DNA binding 2, HLH protein | T cell regulation |
| ID3 | inhibitor of DNA binding 3, HLH protein | T cell regulation |
| IL15 | interleukin 15 | T cell regulation |
| IL18 | interleukin 18 | T cell regulation |
| IL22 | interleukin 22 | T cell regulation |
| M6PR | mannose-6-phosphate receptor, cation dependent | T cell regulation |
| CBLB | Cbl proto-oncogene B | T cell receptor signaling |
| CD40LG | CD40 ligand | T cell receptor signaling |
| IFNA17 | interferon alpha 17 | T cell receptor signaling |
| NFKBIA | NFKB inhibitor alpha | T cell receptor signaling |
| PTPN6 | protein tyrosine phosphatase non-receptor type 6 | T cell receptor signaling |
| ZAP70 | zeta chain of T cell receptor associated protein kinase 70kDa | T cell receptor signaling |
| EGR2 | early growth response 2 | T cell differentiation |
| LEXM | lymphocyte expansion molecule | T cell differentiation |
| BUB1 | BUB1 mitotic checkpoint serine/threonine kinase | Proliferation |
| CCNB2 | cyclin B2 | Proliferation |
| CDK1 | cyclin-dependent kinase 1 | Proliferation |
| CDKN3 | cyclin-dependent kinase inhibitor 3 | Proliferation |
| FOXM1 | forkhead box M1 | Proliferation |
| KIAA0101 | KIAA0101 | Proliferation |
| MAD2L1 | MAD2 mitotic arrest deficient-like 1 (yeast) | Proliferation |
| MELK | maternal embryonic leucine zipper kinase | Proliferation |
| MKI67 | marker of proliferation Ki-67 | Proliferation |
| TOP2A | topoisomerase (DNA) II alpha | Proliferation |
| FOXO1 | forkhead box O1 | PD-1 signaling, tumor marker |
| HIF1A | hypoxia inducible factor 1 alpha subunit | PD-1 signaling, tumor marker |
| MTOR | mechanistic target of rapamycin | PD-1 signaling, tumor marker |
| PIK3CA | phosphatidylinositol-4,5-bisphosphate 3-kinase catalytic subunit alpha | PD-1 signaling, tumor marker |
| PIK3CD | phosphatidylinositol-4,5-bisphosphate 3-kinase catalytic subunit delta | PD-1 signaling, tumor marker |
| PTEN | phosphatase and tensin homolog | PD-1 signaling, tumor marker |
| PTPN11 | protein tyrosine phosphatase non-receptor type 11 | PD-1 signaling, tumor marker |
| NFATC1 | nuclear factor of activated T-cells 1 | PD-1 signaling |
| PRDM1 | PR domain 1 | PD-1 signaling |
| KIR2DL2 | killer cell immunoglobulin like receptor, two Ig domains and long cytoplasmic tail 2 | NK cell marker |
| KIR2DL3 | killer cell immunoglobulin like receptor, two Ig domains and long cytoplasmic tail 3 | NK cell marker |
| NCR1 | natural cytotoxicity triggering receptor 1 | NK cell marker |
| NCR3 | natural cytotoxicity triggering receptor 3 | NK cell marker |
| B3GAT1 | beta-1,3-glucuronyltransferase 1 | NK activation |
| FCGR3B | Fc fragment of IgG receptor IIIb | NK activation |
| GNLY | granulysin | NK activation |
| KLRB1 | killer cell lectin like receptor B1 | NK activation |
| KLRF1 | killer cell lectin like receptor F1 | NK activation |
| KLRG1 | killer cell lectin like receptor G1 | NK activation |
| KLRK1 | killer cell lectin like receptor K1 | NK activation |
| PRF1 | perforin 1 | NK activation |
| CA4 | carbonic anhydrase 4 | Neutrophil |
| DGAT2 | diacylglycerol O-acyltransferase 2 | Neutrophil |
| KREMEN1 | kringle containing transmembrane protein 1 | Neutrophil |
| LRG1 | leucine rich alpha-2-glycoprotein 1 | Neutrophil |
| PYGL | phosphorylase, glycogen, liver | Neutrophil |
| FUT4 | fucosyltransferase 4 | Myeloid marker, stem cell |
| S100A8 | S100 calcium binding protein A8 | Myeloid marker, MDSC |
| S100A9 | S100 calcium binding protein A9 | Myeloid marker, MDSC |
| ARG1 | arginase 1 | Myeloid marker |
| CD33 | CD33 molecule | Myeloid marker |
| CEACAM8 | carcinoembryonic antigen related cell adhesion molecule 8 | Myeloid marker |
| MPO | myeloperoxidase | Myeloid marker |
| AIF1 | allograft inflammatory factor 1 | Macrophage |
| ALOX15B | arachidonate 15-lipoxygenase type B | Macrophage |
| CD163 | CD163 molecule | Macrophage |
| CD68 | CD68 molecule | Macrophage |
| FCGR3A | Fc fragment of IgG receptor IIIa | Macrophage |
| CCL18 | C-C motif chemokine ligand 18 | Lymphocyte infiltrate |
| CCL2 | C-C motif chemokine ligand 2 | Lymphocyte infiltrate |
| CCL21 | C-C motif chemokine ligand 21 | Lymphocyte infiltrate |
| CCL3 | C-C motif chemokine ligand 3 | Lymphocyte infiltrate |
| CCL4 | C-C motif chemokine ligand 4 | Lymphocyte infiltrate |
| CCL5 | C-C motif chemokine ligand 5 | Lymphocyte infiltrate |
| CCR5 | C-C motif chemokine receptor 5 | Lymphocyte infiltrate |
| CD2 | CD2 molecule | Lymphocyte infiltrate |
| CD37 | CD37 molecule | Lymphocyte infiltrate |
| CD52 | CD52 molecule | Lymphocyte infiltrate |
| CD63 | CD63 molecule | Lymphocyte infiltrate |
| CORO1A | coronin 1A | Lymphocyte infiltrate |
| CTSS | cathepsin S | Lymphocyte infiltrate |
| CX3CR1 | C-X3-C motif chemokine receptor 1 | Lymphocyte infiltrate |
| CXCR4 | C-X-C motif chemokine receptor 4 | Lymphocyte infiltrate |
| CXCR6 | C-X-C motif chemokine receptor 6 | Lymphocyte infiltrate |
| FCER1G | Fc fragment of IgE receptor Ig | Lymphocyte infiltrate |
| FYB | FYN binding protein | Lymphocyte infiltrate |
| GZMA | granzyme A | Lymphocyte infiltrate |
| GZMB | granzyme B | Lymphocyte infiltrate |
| GZMH | granzyme H | Lymphocyte infiltrate |
| GZMK | granzyme K | Lymphocyte infiltrate |
| IGSF6 | immunoglobulin superfamily member 6 | Lymphocyte infiltrate |
| IL10RA | interleukin 10 receptor subunit alpha | Lymphocyte infiltrate |
| IL2RG | interleukin 2 receptor subunit gamma | Lymphocyte infiltrate |
| ITGB2 | integrin subunit beta 2 | Lymphocyte infiltrate |
| JAML | junction adhesion molecule like | Lymphocyte infiltrate |
| LAMP1 | lysosomal associated membrane protein 1 | Lymphocyte infiltrate |
| LAPTM5 | lysosomal protein transmembrane 5 | Lymphocyte infiltrate |
| LILRB2 | leukocyte immunoglobulin like receptor B2 | Lymphocyte infiltrate |
| LY9 | lymphocyte antigen 9 | Lymphocyte infiltrate |
| NKG7 | natural killer cell granule protein 7 | Lymphocyte infiltrate |
| PTPN7 | protein tyrosine phosphatase non-receptor type 7 | Lymphocyte infiltrate |
| PTPRC | protein tyrosine phosphatase receptor type C | Lymphocyte infiltrate |
| SAMHD1 | SAM and HD domain containing deoxynucleoside triphosphate triphosphohydrolase 1 | Lymphocyte infiltrate |
| SIT1 | signaling threshold regulating transmembrane adaptor 1 | Lymphocyte infiltrate |
| SLAMF8 | SLAM family member 8 | Lymphocyte infiltrate |
| SRGN | serglycin | Lymphocyte infiltrate |
| TAGAP | T-cell activation RhoGTPase activating protein | Lymphocyte infiltrate |
| TARP | TCR gamma alternate reading frame protein | Lymphocyte infiltrate |
| TLR8 | toll like receptor 8 | Lymphocyte infiltrate |
| TNFAIP8 | TNF alpha induced protein 8 | Lymphocyte infiltrate |
| TYROBP | TYRO protein tyrosine kinase binding protein | Lymphocyte infiltrate |
| IKZF1 | IKAROS family zinc finger 1 | Lymphocyte development |
| IKZF2 | IKAROS family zinc finger 2 | Lymphocyte development |
| IKZF4 | IKAROS family zinc finger 4 | Lymphocyte development |
| SH2D1A | SH2 domain containing 1A | Lymphocyte activation |
| SH2D1B | SH2 domain containing 1B | Lymphocyte activation |
| ITGAL | integrin subunit alpha L | Leukocyte migration |
| ITGAM | integrin subunit alpha M | Leukocyte migration |
| ITGB7 | integrin subunit beta 7 | Leukocyte migration |
| SELL | selectin L | Leukocyte migration |
| VCAM1 | vascular cell adhesion molecule 1 | Leukocyte migration |
| LILRB1 | leukocyte immunoglobulin like receptor B1 | Leukocyte inhibition |
| LST1 | leukocyte specific transcript 1 | Leukocyte inhibition |
| DDX58 | DEXD/H-box helicase 58 | Interferon signaling |
| IFI35 | interferon induced protein 35 | Interferon signaling |
| IFI44L | interferon induced protein 44 like | Interferon signaling |
| IFI6 | interferon alpha inducible protein 6 | Interferon signaling |
| IRF4 | interferon regulatory factor 4 | Interferon signaling |
| MX1 | MX dynamin like GTPase 1 | Interferon signaling |
| OAS2 | 2'-5'-oligoadenylate synthetase 2 | Interferon signaling |
| OAS3 | 2'-5'-oligoadenylate synthetase 3 | Interferon signaling |
| AXL | AXL receptor tyrosine kinase | Innate immune response |
| C1QA | complement component 1 q subcomponent A chain | Innate immune response |
| C1QB | complement component 1 q subcomponent B chain | Innate immune response |
| DMBT1 | deleted in malignant brain tumors 1 | Innate immune response |
| IFIH1 | interferon induced with helicase C domain 1 | Innate immune response |
| LCN2 | lipocalin 2 | Innate immune response |
| LYZ | lysozyme | Innate immune response |
| MAPK14 | mitogen-activated protein kinase 14 | Innate immune response |
| MIF | macrophage migration inhibitory factor | Innate immune response |
| NOS2 | nitric oxide synthase 2 | Innate immune response |
| TLR7 | toll like receptor 7 | Innate immune response |
| ABCF1 | ATP binding cassette subfamily F member 1 | Housekeeping |
| G6PD | glucose-6-phosphate dehydrogenase | Housekeeping |
| GUSB | glucuronidase beta | Housekeeping |
| HMBS | hydroxymethylbilane synthase | Housekeeping |
| LMNA | lamin A/C | Housekeeping |
| LRP1 | LDL receptor related protein 1 | Housekeeping |
| POLR2A | polymerase (RNA) II subunit A | Housekeeping |
| SDHA | succinate dehydrogenase complex flavoprotein subunit A | Housekeeping |
| TBP | TATA-box binding protein | Housekeeping |
| TFRC | transferrin receptor | Housekeeping |
| TUBB | tubulin beta class I | Housekeeping |
| BATF | basic leucine zipper ATF-like transcription factor | Helper T cells |
| CCR2 | C-C motif chemokine receptor 2 | Helper T cells |
| CD4 | CD4 molecule | Helper T cells |
| GATA3 | GATA binding protein 3 | Helper T cells |
| IL17A | interleukin 17A | Helper T cells |
| RORC | RAR related orphan receptor C | Helper T cells |
| STAT4 | signal transducer and activator of transcription 4 | Helper T cells |
| STAT6 | signal transducer and activator of transcription 6 | Helper T cells |
| CD27 | CD27 molecule | Drug target |
| CD40 | CD40 molecule | Drug target |
| CD70 | CD70 molecule | Drug target |
| CTLA4 | cytotoxic T-lymphocyte associated protein 4 | Drug target |
| IDO1 | indoleamine 2,3-dioxygenase 1 | Drug target |
| IL10 | interleukin 10 | Drug target |
| IL12A | interleukin 12A | Drug target |
| IL12B | interleukin 12B | Drug target |
| IL2 | interleukin 2 | Drug target |
| KIR2DL1 | killer cell immunoglobulin like receptor, two Ig domains and long cytoplasmic tail 1 | Drug target |
| KLRD1 | killer cell lectin like receptor D1 | Drug target |
| LAG3 | lymphocyte activating 3 | Drug target |
| MS4A1 | membrane spanning 4-domains A1 | Drug target |
| PDCD1 | programmed cell death 1 | Drug target |
| PMEL | premelanosome protein | Drug target |
| SLAMF7 | SLAM family member 7 | Drug target |
| STAT3 | signal transducer and activator of transcription 3 | Drug target |
| TLR9 | toll like receptor 9 | Drug target |
| TNFRSF18 | tumor necrosis factor receptor superfamily member 18 | Drug target |
| TNFRSF4 | tumor necrosis factor receptor superfamily member 4 | Drug target |
| TNFRSF9 | tumor necrosis factor receptor superfamily member 9 | Drug target |
| CD14 | CD14 molecule | Dendritic cell, macrophage |
| CD209 | CD209 molecule | Dendritic cell, macrophage |
| CMKLR1 | chemerin chemokine-like receptor 1 | Dendritic cell, macrophage |
| IL17F | interleukin 17F | Dendritic cell, macrophage |
| IL23A | interleukin 23 subunit alpha | Dendritic cell, macrophage |
| MRC1 | mannose receptor C type 1 | Dendritic cell, macrophage |
| CLEC4C | C-type lectin domain family 4 member C | Dendritic cell |
| HERC6 | HECT and RLD domain containing E3 ubiquitin protein ligase family member 6 | Dendritic cell |
| IL3RA | interleukin 3 receptor subunit alpha | Dendritic cell |
| ITGAX | integrin subunit alpha X | Dendritic cell |
| NRP1 | neuropilin 1 | Dendritic cell |
| TLR3 | toll like receptor 3 | Dendritic cell |
| ZBTB46 | zinc finger and BTB domain containing 46 | Dendritic cell |
| CCR1 | C-C motif chemokine receptor 1 | Cytokine signaling |
| CSF1R | colony stimulating factor 1 receptor | Cytokine signaling |
| CSF2RB | colony stimulating factor 2 receptor beta common subunit | Cytokine signaling |
| CXCL8 | C-X-C motif chemokine ligand 8 | Cytokine signaling |
| HGF | hepatocyte growth factor | Cytokine signaling |
| IFIT2 | interferon induced protein with tetratricopeptide repeats 2 | Cytokine signaling |
| IL13 | interleukin 13 | Cytokine signaling |
| IL1A | interleukin 1 alpha | Cytokine signaling |
| IL21 | interleukin 21 | Cytokine signaling |
| IL2RA | interleukin 2 receptor subunit alpha | Cytokine signaling |
| IL4 | interleukin 4 | Cytokine signaling |
| IL6 | interleukin 6 | Cytokine signaling |
| IL7 | interleukin 7 | Cytokine signaling |
| STAT5A | signal transducer and activator of transcription 5A | Cytokine signaling |
| TNFSF9 | tumor necrosis factor superfamily member 9 | Cytokine signaling |
| CCL17 | C-C motif chemokine ligand 17 | Chemokine signaling |
| CCL20 | C-C motif chemokine ligand 20 | Chemokine signaling |
| CCL22 | C-C motif chemokine ligand 22 | Chemokine signaling |
| CCR4 | C-C motif chemokine receptor 4 | Chemokine signaling |
| CCR6 | C-C motif chemokine receptor 6 | Chemokine signaling |
| CXCL1 | C-X-C motif chemokine ligand 1 | Chemokine signaling |
| CXCR2 | C-X-C motif chemokine receptor 2 | Chemokine signaling |
| CXCR3 | C-X-C motif chemokine receptor 3 | Chemokine signaling |
| NCF1 | neutrophil cytosolic factor 1 | Chemokine signaling |
| VEGFA | vascular endothelial growth factor A | Chemokine signaling |
| ADORA2A | adenosine A2a receptor | Checkpoint pathway |
| BTLA | B and T lymphocyte associated | Checkpoint pathway |
| C10orf54 | chromosome 10 open reading frame 54 | Checkpoint pathway |
| CD160 | CD160 molecule | Checkpoint pathway |
| CD244 | CD244 molecule | Checkpoint pathway |
| CD274 | CD274 molecule | Checkpoint pathway |
| CD276 | CD276 molecule | Checkpoint pathway |
| CD28 | CD28 molecule | Checkpoint pathway |
| CD48 | CD48 molecule | Checkpoint pathway |
| CD69 | CD69 molecule | Checkpoint pathway |
| CD80 | CD80 molecule | Checkpoint pathway |
| CD86 | CD86 molecule | Checkpoint pathway |
| CEACAM1 | carcinoembryonic antigen related cell adhesion molecule 1 | Checkpoint pathway |
| ENTPD1 | ectonucleoside triphosphate diphosphohydrolase 1 | Checkpoint pathway |
| EOMES | eomesodermin | Checkpoint pathway |
| HAVCR2 | hepatitis A virus cellular receptor 2 | Checkpoint pathway |
| ICOS | inducible T-cell costimulator | Checkpoint pathway |
| ICOSLG | inducible T-cell costimulator ligand | Checkpoint pathway |
| IDO2 | indoleamine 2,3-dioxygenase 2 | Checkpoint pathway |
| NT5E | 5'-nucleotidase ecto | Checkpoint pathway |
| PDCD1LG2 | programmed cell death 1 ligand 2 | Checkpoint pathway |
| PVR | poliovirus receptor | Checkpoint pathway |
| TDO2 | tryptophan 2,3-dioxygenase | Checkpoint pathway |
| TGFB1 | transforming growth factor beta 1 | Checkpoint pathway |
| TNF | tumor necrosis factor | Checkpoint pathway |
| TNFRSF14 | tumor necrosis factor receptor superfamily member 14 | Checkpoint pathway |
| TNFSF14 | tumor necrosis factor superfamily member 14 | Checkpoint pathway |
| TNFSF18 | tumor necrosis factor superfamily member 18 | Checkpoint pathway |
| TNFSF4 | tumor necrosis factor superfamily member 4 | Checkpoint pathway |
| VTCN1 | V-set domain containing T cell activation inhibitor 1 | Checkpoint pathway |
| CD79A | CD79a molecule | B cell receptor signaling |
| CD79B | CD79b molecule | B cell receptor signaling |
| FAS | Fas cell surface death receptor | B cell receptor signaling |
| CD19 | CD19 molecule | B cell marker |
| CD22 | CD22 molecule | B cell marker |
| FCGR1A | Fc fragment of IgG receptor Ia | B cell marker |
| FCGR2B | Fc fragment of IgG receptor IIb | B cell marker |
| FCRLA | Fc receptor like A | B cell marker |
| JCHAIN | joining chain of multimeric IgA and IgM | B cell marker |
| NTN3 | netrin 3 | B cell marker |
| POU2AF1 | POU class 2 associating factor 1 | B cell marker |
| SKAP2 | src kinase associated phosphoprotein 2 | B cell marker |
| TNFRSF17 | tumor necrosis factor receptor superfamily member 17 | B cell marker |
| TNFSF13B | tumor necrosis factor superfamily member 13b | B cell marker |
| BCL2 | B-cell CLL/lymphoma 2 | Apoptosis |
| BCL2L11 | BCL2 like 11 | Apoptosis |
| GADD45GIP1 | GADD45G interacting protein 1 | Apoptosis |
| TNFSF10 | tumor necrosis factor superfamily member 10 | Apoptosis |
| CD74 | CD74 molecule | Antigen processing |
| HLA-A | major histocompatibility complex, class I, A | Antigen processing |
| HLA-B | major histocompatibility complex, class I, B | Antigen processing |
| HLA-C | major histocompatibility complex, class I, C | Antigen processing |
| HLA-DMA | major histocompatibility complex, class II, DM alpha | Antigen processing |
| HLA-DMB | major histocompatibility complex, class II, DM beta | Antigen processing |
| HLA-DOA | major histocompatibility complex, class II, DO alpha | Antigen processing |
| HLA-DOB | major histocompatibility complex, class II, DO beta | Antigen processing |
| HLA-DPA1 | major histocompatibility complex, class II, DP alpha 1 | Antigen processing |
| HLA-DPB1 | major histocompatibility complex, class II, DP beta 1 | Antigen processing |
| HLA-DQA1 | major histocompatibility complex, class II, DQ alpha 1 | Antigen processing |
| HLA-DQA2 | major histocompatibility complex, class II, DQ alpha 2 | Antigen processing |
| HLA-DQB2 | major histocompatibility complex, class II, DQ beta 2 | Antigen processing |
| HLA-DRA | major histocompatibility complex, class II, DR alpha | Antigen processing |
| HLA-DRB1 | major histocompatibility complex, class II, DR beta 1 | Antigen processing |
| HLA-E | major histocompatibility complex, class I, E | Antigen processing |
| HLA-F | major histocompatibility complex, class I, F | Antigen processing |
| HLA-F-AS1 | HLA-F antisense RNA 1 | Antigen processing |
| HLA-G | major histocompatibility complex, class I, G | Antigen processing |
| CD1C | CD1c molecule | Antigen presentation |
| CD1D | CD1d molecule | Antigen presentation |
| CD83 | CD83 molecule | Antigen presentation |
| ADGRE5 | adhesion G protein-coupled receptor E5 | Adhesion, migration |
| CD226 | CD226 molecule | Adhesion, migration |
| CD38 | CD38 molecule | Adhesion, migration |
| CD44 | CD44 molecule (Indian blood group) | Adhesion, migration |
| CD47 | CD47 molecule | Adhesion, migration |
| CD53 | CD53 molecule | Adhesion, migration |
| IGF1R | insulin like growth factor 1 receptor | Adhesion, migration |
| ITGA1 | integrin subunit alpha 1 | Adhesion, migration |
| ITGAE | integrin subunit alpha E | Adhesion, migration |
| ITGB1 | integrin subunit beta 1 | Adhesion, migration |
| MADCAM1 | mucosal vascular addressin cell adhesion molecule 1 | Adhesion, migration |
| NCAM1 | neural cell adhesion molecule 1 | Adhesion, migration |
| NECTIN2 | nectin cell adhesion molecule 2 | Adhesion, migration |
| PECAM1 | platelet and endothelial cell adhesion molecule 1 | Adhesion, migration |

**Supplemental Table S5: Cancer types in the analysis of TCGA PanCancer Atlas cohort (N = 10,953)**

| Cancer types | Number of samples | Percentage of samples |
| --- | --- | --- |
| Breast cancer | 1084 | 9.9% |
| Non-small cell lung cancer | 1053 | 9.6% |
| Esophagogastric cancer | 622 | 5.7% |
| Colorectal cancer | 594 | 5.4% |
| Glioblastoma | 592 | 5.4% |
| Endometrial cancer | 586 | 5.3% |
| Ovarian epithelial tumor | 585 | 5.3% |
| Head and neck cancer | 523 | 4.8% |
| Glioma | 514 | 4.7% |
| Renal clear cell carcinoma | 512 | 4.7% |
| Thyroid cancer | 500 | 4.6% |
| Prostate cancer | 494 | 4.5% |
| Melanoma | 448 | 4.1% |
| Bladder cancer | 411 | 3.7% |
| Hepatobiliary cancer | 372 | 3.4% |
| Renal non-clear cell carcinoma | 348 | 3.2% |
| Cervical cancer | 297 | 2.7% |
| Sarcoma | 255 | 2.3% |
| Leukemia | 200 | 1.8% |
| Pancreatic cancer | 184 | 1.7% |
| Pheochromocytoma | 147 | 1.3% |
| Thymic epithelial tumor | 123 | 1.1% |
| Adrenocortical carcinoma | 92 | 0.8% |
| Pleural mesothelioma | 87 | 0.8% |
| Non-seminomatous germ cell tumor | 86 | 0.8% |
| Ocular melanoma | 80 | 0.7% |
| Seminoma | 63 | 0.6% |
| Mature b-cell neoplasms | 48 | 0.4% |
| Cholangiocarcinoma | 36 | 0.3% |
| Miscellaneous neuroepithelial tumor | 31 | 0.3% |

**Supplementary Table S6. Association of high CD40 RNA expression with high CD28 or GITR RNA expression, stratified by CD4/CD8 expression (N= 514)**

| **Characteristics** | | | **Univariable analysis in patients with high CD4/8 expression**  **(n = 150)** | | **Univariable analysis in patients with low/moderate CD4/8 expression**  **(n = 300)** | |
| --- | --- | --- | --- | --- | --- | --- |
|  |  |  |  |  |  |  |
| Name | Condition | Proportion of tumors with high CD40  expression | Odds Ratio  (95% CI) | *P* value | Odds Ratio  (95% CI) | *P* value |
| CD28 RNA level | High | 42% [43/102] | 2.0 (1.02–3.9) | **0.043** | 2.2 (0.83–5.4) | 0.088 |
|  | Low/Moderate | 17% [71/412] |  |  |  |  |
| GITR  (TNFRSF18)  RNA level | High | 39% [39/99] | 3.0 (1.4–6.3) | **0.003** | 2.2 (1.1–4.3) | **0.027** |
|  | Low/Moderate | 18% [75/415] |  |  |  |  |

^a^Definition of RNA expression is high = 75th–100th percentile RNA rank, moderate = 25th–74th, and low = 0–24th percentile rank value score.

**Supplemental Table S7. Analysis of factors associated with overall survival among patients who received immunotherapy at some point (n = 217)**

| **Characteristics** | **Univariable** | | **Multivariable** | |
| --- | --- | --- | --- | --- |
|  |  |  |  |  |
|  | Median OS months | *P* value | Hazard Ratio  (95% CI) | *P* value |
| Age (years) ≥61 (n = 116) vs. <61 (n = 101) | 16.8 vs. 16.2 | 0.77 |  |  |
|  |  |  |  |  |
| Sex, men (n = 95) vs. female (n = 122) | 15.0 vs. 18.0 | 0.61 |  |  |
|  |  |  |  |  |
| PD-L1 IHC, CPS ≥1 (n = 87) vs. Negative (n = 130) | 15.0 vs. 16.4 | 0.66 |  |  |
|  |  |  |  |  |
| TMB (mutations/Megabase), ≥10 (n = 22) vs. <10 (n = 169)^a^ | 17.9 vs. 11.4 | 0.055 |  |  |
| PD-1, High (n = 44) vs. Low/Moderate (n = 173) | 43.6 vs. 15.0 | 0.0040 | 0.80 (0.43–1.51) | 0.50 |
| PD-L1, High (n = 36) vs. Low/Moderate (n = 181) | 25.7 vs. 15.5 | 0.054 |  |  |
| PD-L2, High (n = 51) vs. Low/Moderate (n = 166) | 22.5 vs. 14.6 | 0.020 | 0.84 (0.51–1.36) | 0.48 |
| CTLA-4, High (n = 41) vs. Low/Moderate (n = 176) | 39.6 vs. 14.6 | 0.0021 | 0.66 (0.36–1.29) | 0.25 |
| LAG3, High (n = 51) vs. Low/Moderate (n = 166) | 23.2 vs. 14.9 | 0.0031 | 0.69 (0.41–1.16) | 0.16 |
| CD40, High (n = 44) vs. Low/Moderate (n = 173) | 20.4 vs. 16.2 | 0.040 | 0.83 (0.47–1.36) | 0.41 |

^a^ Among 217 patients, TMB were available in 191 patients.

Variables with *P* ≤ 0.05 from univariate analyses were included for multivariate analysis.

**Supplemental Table S8. Univariable Cox proportional hazards analysis of CD40 RNA expression as a continuous variable for survival outcomes (N = 514)**

| Cohort | n | Outcome | Hazard ratio  (per 1-unit percentile increase) | 95% CI | *P* value |
| --- | --- | --- | --- | --- | --- |
| All cancer patients who never received immunotherapy | 272 | Overall survival from metastatic disease | 0.997 | 0.991–1.003 | 0.36 |
| All immunotherapy-treated patients | 217 | Overall survival from ICI start | 0.991 | 0.985–0.997 | **0.003** |
| All immunotherapy-treated patients | 217 | Progression-free survival from ICI start | 0.995 | 0.990–1.000 | 0.052 |

Abbreviations: CI, confidence interval; ICI, immune checkpoint inhibitor.
